# A Monte Carlo simulation approach for estimating the health and economic impact of interventions provided at a student-run clinic

**DOI:** 10.1371/journal.pone.0189718

**Published:** 2017-12-28

**Authors:** Daniel J. Arenas, Elle Lett, Heather Klusaritz, Anne M. Teitelman

**Affiliations:** 1 Perelman School of Medicine, University of Pennsylvania, Philadelphia, PA, United States of America; 2 Department of Family Medicine and Community Health, Perelman School of Medicine, University of Pennsylvania, Philadelphia, PA, United States of America; 3 Department of Family and Community Health, School of Nursing, University of Pennsylvania, Philadelphia, PA, United States of America; 4 Department of Obstetrics and Gynecology, Perelman School of Medicine, University of Pennsylvania, Philadelphia, PA, United States of America; TNO, NETHERLANDS

## Abstract

**Background:**

Student Run Clinics (SRCs) are a common aspect of medical education, present at more than half of US medical schools, and noted for providing care to communities that might otherwise lack access, including the uninsured and underinsured. To date, few studies have rigorously quantified the health and economic benefits of SRCs, and the present study remedies that.

**Methods and findings:**

We used Monte Carlo simulations to estimate the health impact of common preventive health interventions applied to individuals in quality-adjusted life-years (QALYs). We then used those measurements to estimate the health and economic impact of United Community Clinic (UCC), a student-run clinic in Philadelphia, PA. We found that with an annual operating budget of $50,000, UCC saves 6.5 QALYs, corresponding to over $850,000 saved.

**Conclusions:**

Using Monte Carlo simulation methods, the health and economic impact of SRCs can be reasonably estimated to demonstrate the utility of SRCs and justify their growing importance in the healthcare delivery landscape of the US.

## Introduction

More than half of medical schools in the country have student-run clinics (SRCs), and a great fraction of the providers are students in their preclinical years [1]. Further, these clinics are increasingly adopting interprofessional models with as many as 76% of SRCs operated by students from at least 2 or more healthcare related professional schools, including nursing, pharmacy, and social work, in addition to medicine [2]. These numbers show that SRCs are becoming an integral part of US medical education, and the benefits are numerous. SRCs expose students to clinical experience before standard hospital rotation, help drive the AAMC mission to improve curricula of health delivery to underserved populations [3], and, along with other innovative healthcare provisions such as mobile health clinics, improve overall student, patient, and provider satisfaction [4–7]. Further, there is evidence that training students in marginalized populations makes them more likely to continue serving these populations after they complete their training [8], suggesting that SRCs may contribute to reducing the primary care shortage currently facing healthcare in the US [9].

SRCs also play a small, but critical role in the national effort to increase preventive healthcare [10]. These efforts are important because there is strong evidence that preventive services save a considerable amount of life-years and improve quality of life thereby reducing national healthcare expenditures and expanding the productive workforce [11,12]. Despite these potentially substantial health and economic benefits of SRCs, to our knowledge there have been no studies quantifying their impact. Here, we propose a novel application of Monte Carlo simulations to estimating the health impact of selected preventive health intervention for individual patients and use the estimates to quantify the health and economic impact of United Community Clinic (UCC), an SRC in Philadelphia, PA.

### Measures of impact

A common way to quantify the health impact or clinically preventable burden (CPB) of a screen or intervention is to calculate the gain of quality-adjusted life-years (QALYs). Many interventions have the clear desirable goal of increasing the life of a patient, but they can also prevent health complications that would otherwise decrease quality of life [13]. Therefore, QALYs reflect the fact that it is also worthwhile to prevent non-fatal complications. The decreases in the quality of life caused by a disease are quantified by using a health utility index, where the weights of disabilities and conditions are obtained from surveys of general populations [14]. The utility of the QALYs is clear in its capability to compare interventions across different diseases; they are an attempt to use empirical evidence for decisions that involve allocation of health resources [13,15]. Most studies report estimates of QALYs for applying an intervention to a birth cohort of four million interventions [16–20]. For the purposes of SRCs, however, which serve a much smaller population; it is more practical to estimate the QALYs per intervention for a single individual, as is done in the present study. Converting the QALYs gained to costs saved is nontrivial and involves estimating healthcare costs, costs to patients (wages lost, etc), and costs to society for the disease associated with a given intervention. Here we use estimates derived from the work of Ubel *et al*. who describe the process of determining costs/QALY in detail [21].

### Description of United Community Clinic

UCC is an interdisciplinary clinic of students in medicine, nursing, dentistry, optometry, social work, and undergraduate studies. The clinic is located in the predominantly African-American, East Parkside Community of Philadelphia, and serves approximately 500 patients from the region and surrounding areas per year. All patients are accepted regardless of insurance status and identification is not required. Most patients do not have a chief concern but are instead requesting physical examinations for PA driver licenses, sports participation, and new jobs. These annual physical exams provide an opportunity to perform preventive medicine and bolster work-force engagement in the form of screenings, simple interventions, and referrals to needed social services. Examples of these services include screenings for blood pressure (BP), blood glucose, high body mass index (BMI), human immunodeficiency virus (HIV), tuberculosis (TB), and dental hygiene. Additionally, through the efforts of integrated social workers, UCC helps uninsured patients obtain insurance and identify at-risk patients that need assistance for housing stability, food safety, addiction-management, and other similar services. Such a diverse array of services is only possible thanks to the inter-disciplinary nature of UCC.

All of these services are offered through an annual budget less than $50,000 which is mostly allocated to medical supplies. The costs are kept low in part due to the volunteerism of students and supervising faculty. Because of the broad range of interventions offered by the clinic, and its comparatively low cost, UCC proves an excellent opportunity to empirically evaluate our Monte Carlo simulation approach to estimate the health and economic impacts of the clinic. We also prospectively estimate the impact of candidate interventions to be added to the services provided by UCC.

## Methods

### Monte Carlo simulations to estimate QALYs/10^3^-interventions

The QALYs gained by an intervention are calculated by using, among other parameters, the change in quality of life of the disease; the time-course of the disease; possible complications or burdens of disease; and the portion of people that develop the burdens (for example, the percentage of patients with hypertension that develop cardiovascular complications). The calculations also use effectiveness in service in follow-up likelihoods, adherence, and risk reduction of developing further complications [17–20,22]. The number of relevant parameters vary substantially depending on the disease and intervention, for example one must consider a large number of associated diseases when calculating the QALYs saved for tobacco screening, but far fewer diseases, most notably pneumonia, when considering influenza vaccines.

As an example, we present detail in the supplementary documents (S2) of the CPB computation flow for the QALYs gained from the influenza intervention (To avoid convoluting the body of the article we have placed the equations in S2 and focus here on the concepts of the CPB calculation). These calculations and input parameters, originally designed by Solberg et al. [20], use a birth cohort of 4,000,000. First, we focus on seniors over 65 years old. We use the influenza-related annual mortality rate per 100,000 as an input parameter (range: 9.4–15.6), and the number of person-years in this age range of the cohort (~50,000,000), to calculate the number of deaths attributable to influenza. The process is repeated for the 50–64 age range. This gives us an estimate of the current mortality burden of the disease for a birth cohort (~85,000 deaths). Since the CPB calculations compare intervention vs no-intervention, we are interested in the theoretical value of the number of mortalities in the birth cohort that would occur if no one were vaccinated. For this we use the efficacy of the annual vaccine in preventing influenza-related mortalities (0.35–0.55), and the current prevalence (0.43–0.72, for ages 65+) of vaccination and estimate that ~110,000 people in the cohort would die as seniors due to influenza-related mortalities. With similar logic, we also calculate the number of hospitalizations and influenza-like illnesses if no one were vaccinated. Second, the intervention that we are studying is offering vaccination to everyone in the birth cohort and must therefore take into account that only a percentage of patients would (or could) accept the vaccine (adherence: 0.75–0.95). Therefore, the effectiveness of the intervention is a combination of the adherence and efficacy of the vaccine. Third, once we know the effectiveness of the vaccine, we can use this value to estimate the impact of the intervention (as a direct comparison to no-intervention) by calculating the number of mortalities in the cohort prevented by the vaccine, and we obtain that approximately 41,000 influenza-related mortalities would be prevented in the cohort. Each mortality receives a full weight of 1 QALY reduction. Similarly we calculate the number of hospitalizations and illnesses (~180,000 and ~2,700,000) that were prevented by offering the vaccine to the whole birth cohort, and assign a QALY reduction (0.20–0.40) for the time that patients would be sick. Adding the QALYs saved from preventing death, hospitalization, and illness gives the final result of the QALYs saved (~300,000). This example of a CPB calculation shows how the error in the estimates of each input parameter would propagate through intermediate results, suggesting the need to assess the precision of the final estimated QALYs saved.

Monte Carlo simulations offer a great opportunity for estimating how the distribution of input parameters affects the distribution of the final result. To perform the simulations for each preventive-medicine intervention, we expanded on CPB calculations performed in the literature. Monte Carlo simulations were only possible if the manuscript with the CPB calculations showed step-by-step explanations of their computations and also provided range or disruption for each input parameter (i.e. adherence, efficacy, etc…). These requirements were satisfied for studies that examined interventions for hypertension screening [18], tobacco screening [17], alcohol misuse screening [19], influenza vaccination [20], and condom distribution [22]. Our Monte Carlo simulations expanded on the previous work by incorporating the error in estimating the input parameters to generate a distribution for the estimated QALYs gained for providing an intervention, and we report the corresponding mean, standard deviation, and quantile intervals for these results. For each intervention, we extracted the input parameters from CPB calculations that provide a range of values found in the literature through systematic reviews [17–20,22]. For example, the CPB calculations by Maciosek et al.[20] report that the efficacy of the influenza vaccine in preventing influenza-like illness can have a range of 0.10–0.30. One limitation of these studies is that they had to draw values from a heterogeneous population of studies and could only provide the maximum and minimum for each parameter. These values are only sufficient to parametrize a uniform distribution for our simulation studies. To determine how robust our methods were to distributional assumptions, we performed a sensitivity analysis, repeating the simulations but using a normal distribution for the input parameters with a mean and variance from the corresponding uniform distribution. Lastly, it should also be stated that because we sample from a uniform distribution that assigns equal probability to all reasonable values reported in the CPB calculations [17–20,22] for each parameter, the quantile intervals for our simulations encompass most reasonable estimates for QALYs saved.

The first step in the Monte Carlo simulations was to design one iteration, based on the CPB calculation, where the QALYs/interventions-individual was calculated based on one set of randomly sampled parameters from the uniform distributions as described above. This process was repeated for 10^6^ iterations. This yielded 10^6^ unique estimates for QALYs/intervention-individual approximating the distribution of possible values given that each estimate is dependent on multiple potentially varying parameters. The results are summarized based on the mean, standard deviation, and 95% quantile intervals which are determined based on the 0.025 and 0.975 quantiles of the simulated distribution (See Table 1). All simulations were performed in Python,[23] and S3 Appendix in the supporting information provides the corresponding code which also contains details on the potentially varying parameters. All graphical analyses are performed in R version 3.4.2.[24]

**Table 1:** QALYs/10^3^-interventions as calculated by Monte Carlo simulations.

Intervention	QALYs (SD) (Per 10^3^ interventions)	95% Quantile Intervals
Tobacco screening and brief counseling^**ǂ**^	1.5 (0.22)	[1.1, 1.9]
Alcohol misuse screening and brief counseling	1.2 (0.47)	[0.51, 2.2]
Hypertension screening and treatment	2.9 (1.0)	[1.4, 5.2]
Free condoms offered to a patient (month's supply)	0.33 (0.12)	[0.12, 0.59]
Influenza vaccine (>50 years)	2.1(0.45)	[1.4, 3.1]
Influenza vaccine (15–49 years)	0.067 (0.022)	[0.030, 0.12]

^ǂ^A conservative estimate of 3% was used for the efficacy of counseling in inducing long-term tobacco quits.

For simplicity, we will report the QALYs gained per 10^3^ interventions. This will improve readability of these values. Additionally, a thousand interventions is a reasonable order of magnitude for the clinic flow of an innovative health care provider such as UCC.

As mentioned above, Monte Carlo simulations could only be performed for interventions for which the literature had manuscripts that provided step-by-step details of the computations and values for input parameters. Some of the interventions had studies that presented the results for QALYs gained for a cohort, but did not have sufficient information to reproduce the study nor allow a parametrized simulation. These interventions are listed in Table 2, and they include obesity screening, depression screening, and others. Although there was not enough information to run a simulation and/or obtain information about the error, the mean of the QALYs/10^3^-interventions were still calculated. S1 provides detail on which studies were explored and how the final results were obtained. Finally, since Monte Carlo simulations could not be performed, we had to approximate the standard deviation of the QALYs/10^3^-interventions. We assumed that the ratio of mean to standard deviation, the coefficient of variation (CV), is similar to all preventive health interventions in this Monte Carlo simulation study.

**Table 2:** Interventions with estimated coefficients of variation.

Intervention	QALYs (SD) (Per 10^3^ interventions)
Obesity screening (adults) and brief intervention	6.0 (2.0)
Depression screening and counseling	0.50 (0.16)
HIV-risk screening	0.27 (0.09)
Syphilis-risk screening	0.030 (0.010)
Diabetes screening and treatment (>25 yr old)	2.0 (0.66)
Hypercholesterolemia screening and treatment	2.5 (0.83)
Colon cancer screening (colonoscopy)	33 (11)
Breast cancer screening and treatment (Mammographies)	17 (5.6)
Pap smears (Cervical cancer screening)	10 (3.3)

Interventions for which Monte Carlo simulations were not possible due to inaccessibility to detailed CPB calculations. S1 of the supplementary documents details how the mean QALYs/10^3^-interventions were calculated. The methodology section explains how and why the standard deviations were approximated as one-third of the mean.

### Estimating the healthcare costs saved by UCC

For each intervention we estimated the cost saved by UCC based on the patient load of the clinic and the cost associated with each QALY saved. For the purpose of this study, we use the estimated minimum cost of associated with each QALY saved proposed by Ubel *et al*. in 2003 [21], $100,000, a value which is decided by taking into account healthcare costs, lost wages, costs to society, and society’s willingness to pay for a QALY. We adjusted this value for inflation for an estimated $132,200/ QALY in 2017 [21]. For universal services, or interventions that all patients at UCC receive, we report the cost saved as the product of the QALYs/10^3^-interventions, the cost saved per QALY, and the patient load at UCC, 500 patients per year, adjusting for a 20% fluctuation in patients between years. For services that are only applied to a subset of individuals based on specific characteristics such as age (for vaccinations) and BMI (for diabetes screening), we estimated the number of individuals who were provided with that service at UCC over the past year. The estimates were: 100 diabetes screenings; 10 mammography referrals; 50 and 5 influenza vaccinations for adults and seniors, respectively. Similarly to the universal screenings, we assumed a 20% fluctuation in patients between years for the estimates.

We estimated the total health impact of the clinic by multiplying the QALYs saved per intervention by the number of interventions performed at UCC. Then, the economic impact of the clinic was obtained by using the dollar value of a QALY. We assume independence when estimating the variance of costs saved per intervention, because the cost per QALY, QALYs/10^3^-interventions, and UCC patient load should be uncorrelated. S3 also offers the Python code for estimating the impact of the clinic based on patient volume.

Because this study did not involve the review of identifiable patient information it did not require IRB approval, because the patient volume was estimated based on the number of records, which remained sealed throughout the study.

## Results

**Fig 1:**
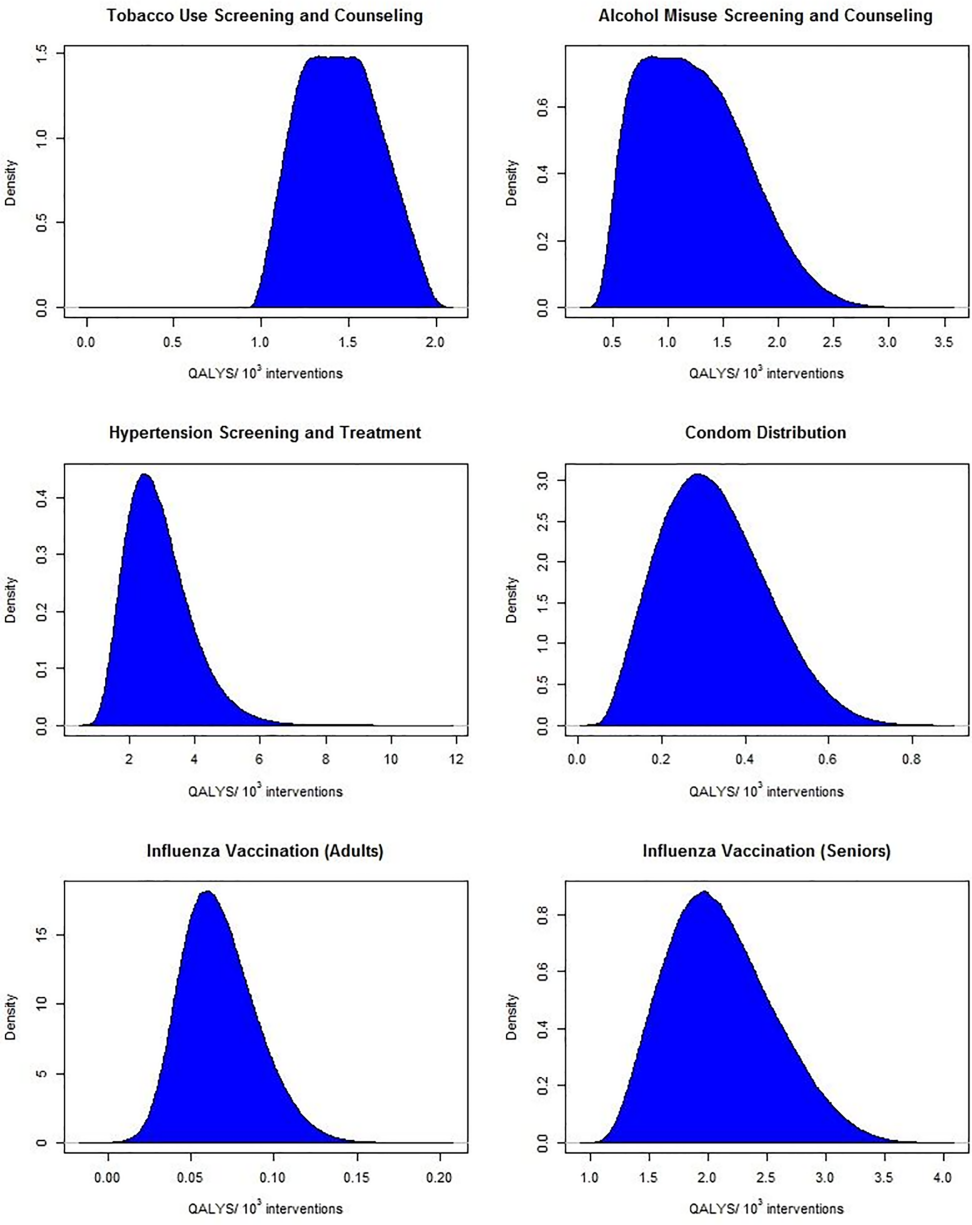
Kernel density estimation plots for QALYs/10^3^-interventions for 10^6^ Monte Carlo simulations.

A total of 10^6^ Monte Carlo simulations were ran to estimate the QALYs/10^3^-interventions for tobacco use screening and brief counseling, alcohol misuse screening and brief counseling, hypertension screening and treatment, condom distribution, influenza vaccinations for individuals aged 15–49 years, and influenza vaccinations for individuals over the age of 50 years. Fig 1 shows kernel density estimates of the simulated distributions for the QALYs/10^3^-interventions for each intervention and Table 1 reports the mean and standard deviation, as well as the 95% quantile intervals (QIs). Hypertension screening had the greatest estimated health impact at 2.9 (95% QI: 1.4, 5.2) QALYs/10^3^-interventions, followed by influenza vaccines in individuals over the age of 50 years old (2.1, 95% QI: 1.4, 3.1). Fewest QALYs/10^3^-interventions were saved by influenza vaccines in individuals 15–49 years old (0.067, 95% QI: 0.030–0.12). Tobacco use screening exhibited the most stable estimates for QALYs/10^3^-interventions with the estimated standard deviation approximately 15% of the mean, compared to alcohol misuse screening which had the least stable estimates with a standard deviation-to-mean ratio of 40%.

Table 2 shows the estimates for QALYs/10^3^-interventions for interventions that did not have enough information in the literature to parametrize a simulation study. These included obesity screenings and brief interventions in adults, depression screening and counseling, HIV and syphilis risk screening, diabetes in individuals over 25 years old and hypercholesterolemia screening and treatment, breast cancer screening and treatment and cervical cancer screening with pap smears. Breast cancer screening and treatment had the highest estimated health impact at 17 QALYs/10^3^-intervention, while syphilis risk screening had the lowest at 0.030 QALYs/10^3^-interventions.

The health and economic impacts for UCC in QALYs saved/year and dollars, respectively, are reported in Table 3 with corresponding standard deviations. As shown in Table 3, obesity screening and brief interventions in adults had the highest health impact at 3.0 QALYs saved per year (SD: 1.2 QALYs/year), corresponding to $390,000 saved per year (SD: $154,000/year). In comparison, syphilis risk screenings were the least impactful, only contributing 0.015 QALYs/year (SD: 0.006 QALYS/year), corresponding to $2,000 saved per year (SD: $770/year). Overall, the UCC clinic saves an estimated 6.55 QALYs/year (SD: 1.70 QALYs/year) corresponding to $851,000 annually (SD: $211,000/year).

**Table 3:** Annual health and economic impact of the preventive health interventions at UCC.

Intervention	QALYs/year (SD)	Thousands of Dollars (SD)
Obesity screening (adults) and brief intervention	3.0 (1.2)	396 (155)
Hypertension screening and treatment	1.45 (0.58)	192 (77)
Tobacco screening and brief counseling	0.75 (0.19)	99 (25)
Alcohol misuse screening and brief counseling	0.60 (0.27)	79 (35)
Free condoms offered to a patient	0.17 (0.070)	22 (9.2)
HIV-risks screening	0.14 (0.05)	18 (7.0)
Syphilis-risks screening	0.015 (0.006)	2.0 (0.78)
Influenza vaccine (>50 years old)*	0.011 (0.003)	1.4 (0.41)
Influenza vaccine (15–45 years old)*	0.0034 (0.0013)	0.44 (0.17)
Diabetes screening and treatment (>25 yr old)*	0.20 (0.08)	26 (10)
Breast cancer screening/treatment (mammographies)*	0.17 (0.07)	22 (8.8)
**Total**	**6.50 (1.37)**	**858 (180)**

Patient volume was approximated as 500 per year.

*Based on an estimated 50 influenza vaccinations for adults, 5 influenza vaccinations for seniors, 100 diabetes screenings, and 10 mammography referrals at UCC in the past year. 20% yearly fluctuations in patient visits, and services offered, were used to estimate the statistics of the final results. 20% fluctuation in the value of the QALY was also used.

Table 4 reports the potential health and economic impact of services that might be offered at UCC in the future, specifically colon cancer screening and referrals, depression screening and treatment, and cholesterol screening and treatment. Based on our estimations, the intervention that will have the greatest impact at UCC would be colon cancer screening which would save approximately 0.33 QALYs/year (SD: 0.13 QALYs/year) corresponding to $43,000 annually (SD: $17,000/year). If all 3 interventions were added to the UCC services, the clinic would save an additional 0.71 QALYs/year (SD: 0.17 QALYs/year) corresponding to $92,000 annually (SD: $22,000/year).

**Table 4:** Annual health and economic impact of potential preventive health interventions at UCC.

Procedures	QALYs/year (SD)	Thousands of Dollars (SD)
Colon cancer screening referrals	0.33 (0.13)	44 (17)
Depression screening and treatment	0.25 (0.10)	33 (13)
Cholesterol screening and treatment	0.13 (0.05)	17 (6.5)
**Total**	**0.71 (0.17)**	**93 (22)**

Based on universal screening for depression (500 patients), 50 cholesterol screenings, and 10 colonoscopy referrals. 20% fluctuations in the number of interventions were considered for the statistical analysis.

## Discussion

Using Monte Carlo simulations we estimated the QALYs saved for an individual for selected preventive health interventions, and applied these estimates to determine the health and economic impact of the United Community Clinic of Philadelphia, PA. We showed that on an annual budget of less than $50,000, UCC’s preventive services save over 6 QALYS/year, corresponding to more than $850,000/year, or a greater than 17-fold annual return on investment (ROI).

To our knowledge, this is the first application of Monte Carlo simulations to estimating the clinically preventable burden of the interventions selected in this study. This method has the benefit of providing statistical information about the final results (i.e. QALYs). We generated a distribution of 10^6^ estimates of QALYS/individual-intervention that allowed us to estimate not only the mean but also the variance and provide precise intervals for values previously reported in the literature [16–20,22]. Further, as shown in S4 of the supplementary documents, the simulation estimates tend to converge as early as 10^4^, suggesting that fewer simulations are necessary to generate accurate estimates.

The Monte Carlo method also appeared to be robust to the choice of distribution for the input parameters. Here we report on results from sampling the parameters from uniform distributions, but when using a normal distribution the estimates and intervals were similar. For example, the tobacco-intervention calculations yielded mean and standard deviations of 1.4 (0.22) and 1.5 (0.22) QALYs/10^3^-interventions, from calculations using uniform distribution and normal distribution inputs, respectively. For the alcohol intervention, both input distributions yielded final results of 1.2 (0.47) QALYs/10^3^-interventions.

The utility of estimating the costs saved by SRCs is high. Such calculations can be used to justify continued or extended financial and personnel support for SRCs, bolster grant applications, and generate community and institutional support. Further, by prospectively estimating the benefit of proposed additions to the services offered by UCC, we help prioritize which potential interventions will provide the greatest impact based on the specific patient load and demographics of UCC. By our method, we estimate that colon cancer screenings referrals for 10 patients would add an additional 0.33 QALYs/year to the health impact of UCC, corresponding to $43,000 in costs saved. This models a framework that other SRCs can adopt to select services for expansion, especially those SRCs with small operating budgets for which resource allocation is critical. We report QALYs/10^3^-interventions which can be readily scaled for estimates of impact in other SRCs. Further, we also furnish the code and detail the methodology in the supporting information so that other clinics might generate their own estimates QALYs/10^3^-interventions for other services that are not explored here. In sum, we hope that this work can contribute to the sustainability of student run clinics by providing a rigorous method of quantifying the benefits they provide to the communities they serve.

## Limitations

This study has several limitations. First, only a subset of the interventions were investigated using Monte Carlo simulations; tobacco, alcohol and hypertension screenings, condom distribution, and influenza vaccinations. Only these had clinically preventable burden estimates in the literature that were detailed enough to support parametrization in our simulations. For the remaining interventions, screenings for obesity, depression, HIV risk, syphilis risk, diabetes, cholesterol, colon cancer, and cervical cancer we estimated the standard deviation as one-third of the mean QALYs reported in the literature, based on our observation that the simulated distributions had coefficients of variation between 15 and 40%. Therefore, our results for these total QALYs and costs saved by these interventions should be conservative. Ideally, all the reported mean and variances of the QALYs/10^3^-interventions values would be obtained from Monte Carlo simulations; however, the current literature did not allow this. These interventions are an important subset of the clinics; it is therefore imperative that we still include these interventions, even if the standard deviation had to be approximated, for they are an integral part of clinics with preventive medicine goals.

This study is also limited to quantifying the impacts of the selected interventions and does not examine other, potentially large impact, contributions from other services, like brief counseling for behavior modifications in diet and exercise. Further, this study does not differentiate between benefits due to the direct action of the SRC from those primarily due to services obtained through specialist referrals. This limitation might suggest that we overestimate the impact of UCC, but, given that we apply a conservative estimate for the financial gains associated with each QALY saved [21], and do not consider all services offered at the clinic, the authors suspect that this overestimation effect is negligible. Furthermore, existing CPB calculations of vaccinations do not estimate the effect of preventing secondary influenza infections of seniors or other populations at risk when we vaccinate adults. Additionally, this study does not consider the costs saved to patients in the SRC model where services are provided free of charge. For example, for an uninsured patient, the approximate cost of a physical exam is $230, which is waived at UCC. Assuming half the exams given by the clinic go to uninsured patients that corresponds to an additional $57,000, saved by the clinic. This limitation also suggests that the impact of SRCs may be underestimated in the present study.

## Conclusions

SRCs are a common aspect of medical education and are noted for providing care to communities that might otherwise lack access, including the uninsured and underinsured. The present study extends the understanding of the utility of SRCs by quantifying health and economic impact and demonstrating that a single clinic can yield as high as a 17-fold return of investment on costs saved to patients and society through preventive health interventions. Further studies should parse the contributions of integrated specialty services and referral systems from contributions directly attributable to SRCs and expand the literature on the benefits of other services, particularly counseling services, offered by SRCs.

These simulation methods and results should also be of useful to other healthcare providers interested in evidence based allocation of resources. This study should also impact the research community by corroborating the importance of studying parameters of disease such as probability of complications, mortality rate, efficacy of treatment, adherence, etc. . . As these values are more precisely estimated in the literature, the findings in this study can be updated to more conclusively assess the impact of SRCs and other innovative healthcare providers.

## Supporting information

S1 Appendix "Details for the calculation of QALYs/10^3^-interventions". (PDF)

S2 Appendix "Equation flow for the clinically preventable burden (CPB) calculations for influenza vaccination of seniors". (PDF)

S3 Appendix "Python code for CPB calculations using Monte Carlo simulations". (ZIP)

S4 Appendix "Convergence of CPB calculations versus the number of Monte Carlo simulations". (ZIP)
