# Supplementary material for: A Monte Carlo simulation approach for estimating the health and economic impact of interventions provided at a student-run clinic
Source: PLoS One. 2017 Dec 28;12(12):e0189718. doi: 10.1371/journal.pone.0189718 (PMC5746244; doi:10.1371/journal.pone.0189718)
Supplement: S1 Appendix — (PDF) [file pone.0189718.s001.pdf]

S1: Details for the calculation of QALYs/10^3-interventions

Preventive medicine improves the expectancy and quality of life of a population.[1] Student run clinics (SRCs), and other innovative health providers, contribute to the overall effort to improve long-term care. Each intervention, such as hypertension screening, tobacco-use counseling, or condom distribution, contributes to the universal effort.

This document shows how to estimate the quality-adjusted life years (QALYs) gained by one preventive-medicine intervention performed at one clinic. Having an estimate of the impact per intervention would be useful to an innovative health provider that wants to estimate the health and economic impact of their clinic. These are not, by any means, the only benefits offered by SRCs since there are benefits to health care that cannot be easily quantified. Nonetheless it is important to quantify the gains that could be reasonably studied. The difficulty and importance of calculating the QALYs per intervention merits some detailed discussion for each screening guideline, and the details are presented in this supplementary material.

I. Screening for Tobacco use and counseling

Studies have shown that smokers gain an average of 6 years of life expectancy when they quit smoking.[2] Some reports claim that frequent counseling is about 23% effective in the long term in inducing ever smokers to quit; this means that smokers save about 1 year of life in average when they are repeatedly motivated to quit tobacco.[3]

1. How this work expands on CPB calculations in the literature by adding Monte Carlo capabilities

In 2006 Solberg et al. prepared a technical report for the National Commission on Prevention Priorities in which they explored the cost efficiency of tobacco-use screening and counseling. The purpose of their work was to compare the cost to the benefit (QALYs gained) of implementing the intervention.[3] The cost of the intervention is outside the scope of this work. Here, we will only focus on the QALYs gained.

In their technical report, Solberg et al. showed the high returns, quantified by QALYs, of brief interventions for smoking cessation.[3] Their manuscript shows very detailed explanations of the CPB calculation flow, and readers are encouraged to read these original manuscripts for additional detail. This type of CPB calculations, which are estimating the impact of having an intervention compared to not having it, require parameters such as the effectiveness for repeated counseling for quitting in a primary care practice,[4,5] the prevalence of tobacco use, and the clinical burden of smoking-attributed morbidities. Examples of smoking-attributed morbidities include the large percentage (at least 70%) of oral, pharyngeal, and lung cancers are attributed to tobacco.[6,7]

The report by Solberg is extremely useful in that they also justify the input parameters of the CPB calculation (i.e. efficacy of counseling, risk increase of morbidities for smokers, etc…). They were

thorough and obtained these input parameters, and even a measure of their uncertainty, from systematic searches of the literature.

One area with room for improvement in their CPB calculation manuscript,[3] and others,[8–11] is a rigorous estimation of the uncertainty of the final values of the calculation (i.e. QALYs gained). The uncertainty in input parameters propagates to results in the middle of the calculation, which are then further used to calculate other quantities down the equation flow; eventually carrying the uncertainty of all input parameters into the final result. In the original work,[3] a sensitivity analysis is presented where different input parameters are sampled to get a very rough estimate of the range of the final values. This is where Monte Carlo simulations, and the statistical analysis of the simulations presented in this work, become extremely useful. In this work we show how we can use distributions of input values to directly obtain a statistical distribution of final values (i.e. QALYs), for which we can quantify the mean, uncertainty, and even 95% quantile intervals. S3 presents all the python code so that future researchers can update input parameter values when appropriate.

2. Obtaining QALYs/intervention from QALYs gained in a cohort

The technical report by Solberg et al. reported that tobacco screening and counseling to a one-year birth cohort in the US (4,000,000 people) would save over 2,500,000 QALYs to the cohort. Here we also expand on the original work by estimating the QALYs gained per screening/intervention. To obtain this number, we assume everyone in the cohort has to be screened every year and that the smokers would be counseled. Therefore every annual physical exam that screens for tobacco is counted as an individual-intervention. To estimate the amount of individual-interventions in the lifetime of the cohort, we can use life tables to show that there are 226,000,000 person-years above 15 years old for a 4,000,000 cohort.[12] Using the 2,500,000 QALYs result from Solberg, we would obtain that every annual physical exam that screens for tobacco, and offers counsel to smokers, results in 0.011 QALYs gained per intervention.

For simplicity, we will from now on report the QALYs gained per 10^3 interventions. A thousand interventions is also a convenient value since this is a reasonable order of magnitude for the annual patient volume of an innovative health care provider.

3. Exploring other work in the literature

There are other manuscripts in the literature that report their results for QALYs gained in the cohort, but unfortunately do not provide detail about the CPB calculation nor the input parameters used. One example is the recent study by Maciosek et al. which reports that increasing the current average of tobacco screening from 50% to 90% would result in significant QALYs gained.[13] Since details of the methodology are not reported,[13] we cannot expand on the work by using Monte Carlo simulations. Nonetheless, the publication is still useful in that we can use their results to estimate the QALYs/10^3-intervention. Their calculation also used a cohort of 4,000,000 people and they demonstrated additional benefits of 460,000 QALYs if 40% of the cohort is screened. Similarly, using annual screening to estimate the amount of interventions in the cohort, we obtain a value of 5.0 QALYs/10^3-interventions.

4. *Effectiveness of counseling and our conservative estimate for the CPB calculation*

CPB calculations of tobacco-use interventions are very unique in that their uncertainties are quite large; the large uncertainty arises from the fact that it is not easy to estimate the efficacy of counseling in inducing long-term quits in smoking. A value of 23% is frequently used in the literature,[3] but authors correctly point out that this value can be as low as 3%, or as high as 60% if repeated counseling is utilized.

In our Monte Carlo simulations, we still followed the CPB calculation flow of Solberg et al.[3] but we used the lowest estimate for the efficacy (3%) of counseling against tobacco use. The Monte Carlo simulations then yielded that 332,000 (50,000) QALYs would be gained in a cohort of 4,000,000, and this corresponded to 1.5 (0.2) QALYs/10^3-interventions.

During these CPB calculations, the other input parameters, such as relative risk of morbidities attributed to smoking, were randomized based on the range provided by Solberg et al.[3] For our Monte Carlo simulations, we used both a uniform distribution (based on the range specified in reference[[3]]), and a normal distribution using the mean and standard deviation obtained from the uniform distribution. The final results for the QALYs/10^3-interventions were identical within 1%. S3 in the supplementary documents provides the python code for the simulations.

5. *Other important results and considerations from the calculation*

A 3% efficacy value for tobacco counseling may sound disheartening to health care providers, but our results show that an efficacy as low as 3% still produces massive gains in clinically preventable burden. For a clinic such as UCC, with patient volume of 500, we are still able to make a 0.75 QALYs impact by simply incorporating tobacco use screening (and intervention to those who report positive). These results should leave no doubt that tobacco counseling patients is worthwhile even if there is only a small chance of helping a patient quit.

II. Screening for alcohol-misuse and providing counseling

Along with tobacco, various reports have placed alcohol screening and counseling as one of the highest-ranked preventive services that can be delivered in a primary care setting.[13–16] Our Monte Carlo simulations follow the CPB calculations from a technical report for the prevention of alcohol morbidity.[8]

1. *Example of the logic behind a CPB calculation*

In Maciosek's CPB calculations for the alcohol-misuse intervention,[8] the authors use a 4,000,000 birth cohort and first calculate the amount of QALYs that would be lost to chronic and acute conditions of alcohol if patients were never counseled against alcohol misuse (~2,600,000). To estimate this number, the authors use as input parameters the current amount of life-years lost to these conditions (~2,000,000), the current delivery of screening and counseling (5-20%), the effectiveness of counseling in changing drinking-behavior (10-35%), the efficacy of behavior-change in reducing acute and chronic

alcohol related conditions (10-50%). Once they know how many QALYs are lost without the intervention, they estimated what would happen if the intervention, screening and counseling if necessary, were to be offered to everyone in the cohort. This next step of the calculation takes into account the adherence to the screening (80-95%) since some patients would decline, the sensitivity of the screening to find alcohol misuse (60-90%), the effectiveness of counseling at changing behavior (10-35%), and the efficacy of behavior change in reducing QALYs lost to alcohol morbidities (~60%). Their total result is that ~176,000 would be gained in the cohort if the intervention is implemented.

2. Our Monte Carlo simulations for the alcohol intervention

Our Monte Carlo simulations are able to utilize the uncertainty of the input parameters (as the full range of reported possible values) and obtain 250,000 [95% QI: 105,000 - 460,000] gained QALYs for the cohort. Using the number of interventions required in the cohort, our final results yielded 1.2 [95%QI: 0.51 – 2.2] QALYs/10^3-interventions. It should be mentioned that the CPB Monte Carlo simulations for the alcohol misuse intervention were performed by using two types of distributions for the input parameters: a uniform and a normal distribution. The results for the average and standard deviation for the distributions were identical (within 1%) for both choices of input distributions. These results corroborate the robustness of the Monte Carlo simulations to the choice of distribution for the input parameters. S3 in the supplementary documents provides the python code for the simulations with detail about the CPB calculation and the variances of the input parameters.

3. Other work in the literature

Other manuscripts with results from CPB calculations, but without methodology details, include Solberg et al. who reported that screening people between 18-54 years old, in a cohort of 4,000,000, and counseling those who are found to misuse alcohol, results in QALY gains of 177,000.[16] To extract the QALYs/10^3-interventions we divide the QALY gains by the amount of interventions in the lifetime of the cohort for the age range specified[12] (206,000,000 person-years) and obtain 0.87 QALYs/10^3-interventions. Analyzing a more recent report by Maciosek et al.[13] yields a similar value of 0.78 QALYs/10^3-interventions. Only the mean values can be extracted since there is not enough information to obtain uncertainty, but these references are still useful in validating the results of our Monte Carlo simulations.

III. Screening for hypertension and providing treatment

1. Our results

Our Monte Carlo simulations for the hypertension intervention was based on the Technical Report by Maciosek et al in 2006,[10] which presented detail in the calculation flow and reported the uncertainties of input parameters such as adherence, follow-up, etc…,[10] Our calculations yielded 657,000 (220,000) QALYs gained for the 4,000,000 cohort. Assuming interventions are performed during annual physical exams, we arrive to 2.9 [95%QI: 1.4 – 5.2] QALYs/10^3-interventions. S3 in the

supplementary documents provides the python code for the simulations with detail about the CPB calculation and the variances of the input parameters.

2. Other results in the literature but without methodology details

Using a 4,000,000 cohort, Coffield et al. reported that a total of 686,000 QALYs would be gained if the patients were screened for hypertension throughout their lifetime.[15] This corresponds to 3.0 QALYs/10^3-interventions. Analyzing a more recent study[10] yields a similar number. The most recent report in the impact of hypertension screening uses a 100,000 cohort,[17] and yields a similar value of 2.7 QALYs/10^3-interventions. Once again, these values in the literature fall in our quantiles and corroborate our results.

3. Results in the literature that differed greatly from our results and others in the literature

It is important to note that our estimates for the QALYs gained per hypertension screening are very different from those used by Oriol et al in their study of mobile health clinics.[18] For hypertension, they used around 0.0897 QALYs gained per individual-intervention (90 QALYs/10^3-interventions). Although their work is very important and novel, it is not clear how they arrived to their values, since they seem to reference a paper by Maciosek et al. in 2006,[14] and this manuscript does not report the additional QALYs gained for hypertension. Perhaps the authors meant to cite a previous paper by the same group in 2001;[15] however, even with this citation the calculation seems incorrect. We can only obtain a value in the same order of magnitude as the 0.0897 value by Oriol et al if we assume that the 686,000 QALYs are gained from the 4,000,000 cohort if each patient only gets screened twice in their lifetime. Therefore, we believe that the QALYs/screening values used by Oriol et al.[18] are the result of a miscalculation resulting from not accounting for the fact that there are multiple annual interventions in the CPB calculations. Unfortunately, many of the values they reported for other screenings and interventions have the same problem and will therefore disagree with our estimates.

IV. Screening and treating for obesity, depression, and STI risk

1. Lack in details of CPB calculations and input parameters

Since we did not find a manuscript that presented step-by-step details of their CPB calculations for obesity, depression, or STI screening, we could not perform Monte Carlo simulations to estimate how the variance of the input parameters affects the variance of the final results. As discussed in the main manuscript, we used 1/3 of the mean as the estimate of the uncertainty.

2. Results in the literature

The long-term benefits of obesity screening and prevention have been shown by several reports, especially for early interventions.[19,20] Analyzing a recent report where the guidelines are to screen the BMI of all patients, and to refer those over 30 to behavioral interventions,[13] yields 6.0 QALYs/10^3-interventions. Similarly, the same cohort would gain 45,000 QALYs if an extra 40% of a 4,000,000 cohort is screened for depression.[13] Assuming that screenings are performed annually, this corresponds to

about 0.50 QALYs/10^3-interventions. As every other screening, a positive result must be accompanied by enhancements of patient care.[21] Unfortunately, it is difficult to refer patients since there are very few free or low-cost resources for the treatment of depression. Therefore, the interventions for depression that an SRC can offer would be less effective than interventions and referrals for other conditions such as obesity.

Coffield et al. showed that assessing the risk of STIs, and providing counseling on reducing risk, would save around 50,000 QALYs in a 4,000,000 cohort.[15] This yields 0.22 QALYs/10^3-interventions, if the interventions are done annually after the patients turn 15 years old.

Unfortunately, as recent as 2010, a significant percentage (~40%) of the US population in the 15-44 age range had never been tested for HIV.[22] And, only a small percentage (~20%) is regularly screened for risk factors and tested.[13] Maciosek et al. showed that increasing this percentage to 90% would result in additional 32,000 QALYs gained over the lifetime of the cohort.[13] To obtain QALYs/individual-intervention from their manuscript, we use 70% of a 4,000,000 cohort, and the number of interventions throughout the 15-44 range, and obtain 0.27 QALYs/10^3-interventions. A similarly analysis for syphilis yields 0.035 QALYs/10^3-interventions.

V. Discreet Condom Distribution

Condom distribution is usually performed discreetly so that patients can obtain them without having to ask the providers. To estimate the health impact of the discreet distribution method, we used a method similar to the one by Bedimo et al.; in their study, they made 33,000,000 condoms free and available to 275,000 people.[11]

Their study reported several important results: a 30% increase in condom use among the target population, and they estimated that about 170 HIV infections were prevented. We performed similar calculations, the python code available in S3 of the supplementary documents, where free condoms are available to a cohort of 500 people for 1 year. Some of the input variables included the amount of sexual partners in one year, the annual frequency of intercourse, the prevalence of HIV in the population, the increase in condom use by making condoms available, etc…[11]

Our method expands on Bedimo's work because of our Monte Carlo simulations allow us to calculate how the uncertainties in the input variables affect the mean and standard deviation of the calculation. Our results show that offering a year's supply of free condoms to 500 patients (1000 people by counting the partner of the sexual act) would prevent 0.17 (0.07) HIV infections. This translates to 1.95 (0.75) gained QALYs. We must take into account that an SRC, such as UCC, only offers about 10 condoms per bag which is, as a conservative estimate, about a month's supply for the average couple.[23] Therefore for 500 patients, who were in a position to pick up free condoms in a discreet matter, we save 0.163 (0.063) QALYs, which correspond to $22,000 ($8,000). Quantifying the impact per interventions, we obtained 0.33 [95%QI: 0.12 – 0.59] QALYs/10^3-interventions. This shows that condom distribution in an SRC is an extremely impactful and cost-efficient intervention. S3 in the supplementary documents provides the python code for the simulations with detail about the CPB calculation.

VI. Influenza vaccinations

1. *Seniors (Ages: 50+)*

In influenza vaccination, the highest impact is obtained for vaccination of patients over 50 years old due to the highest risks in this population. Our Monte Carlo simulations for influenza vaccinations are based on the technical report by Maciosek et al[9] in 2006; we obtained 2.1 [95%QI: 1.4 – 3.1] QALYs/10^3-interventions. S2 provides extensive detail.

Other work in the literature has shown that 100,000 additional QALYs are gained when an extra 45% of a 4,000,000 cohort is vaccinated.[13] This corresponds to 2.1 QALYs/10^3-interventions since there would be 107,000,000 vaccinations in the cohort.[12] Analyzing earlier publications[9] would yield similar results of 2.4 QALYs/10^3-interventions.

2. *Adults(Ages:15-49)*

Influenza vaccination for the 15-49 year old range is not reported in many preventive services since it yields a much lower return. Patients in this age range have much smaller probabilities of contracting the disease and developing complications.[24] Nonetheless, however small, we should still estimate the QALYs/individual-intervention.

We modified the calculations from the vaccination of seniors and adjusted the values for adults. A birth cohort of 4,000,000 people will have 118,000,000 person years in the 15-50 age range.[12] At this range, the annual incidence of influenza is around 0.06,[24] and the vaccine effectiveness in preventing medically-attended flu is around 0.5.[25] Therefore if every person is vaccinated about 3,740,000 cases of flu would be prevented in the cohort. At a duration of a week,[26]26 with a 0.30 QALY weight,[9] this corresponds to 25,000 QALYs gained in 118,000,000 interventions. Our Monte Carlo simulations showed that the final results were very sensitive to the efficacy of the influenza vaccination. Therefore, here we used a conservative estimate for the effectiveness of the influenza vaccine, 0.35,[9] and obtained 8,000 (2,650) QALYs gained in the cohort: 0.067 [95% QI: 0.030 – 0.12] QALYs/10^3-interventions. S3 in the supplementary documents provides the python code for the simulations.

VII. Screening and treatment for diabetes and hypercholesterolemia

A 1998 study by the CDC showed that early screening for diabetes can save an average of 0.35 QALYs per patient.[27] Their calculations assumed a single screening per lifetime. Since SRCs provide one of many annual checkups, it would be more accurate to spread the gains over the number of annual physical exams. In average, a 25 yr old will have 55 annual physical exams throughout their life,[12] and this translates to 6.3 QALYs/10^3-interventions. A more recent report by Hoerger et al.[28] shows 0.12 QALYs as a conservative estimate for the gains from early screening and treatment. If we make the same simple assumption that this gain should be spread over 55 interventions, we obtain 2.0 QALYs/10^3-interventions.

For cholesterol screenings, Dehmer et al. showed that 14,300 QALYs would be saved for a 100,000 cohort if the men were screened for hypercholesterolemia after 35 years old and women after 45 years old.[17] Early treatment (>20 yrs old) were recommended to those with higher risk of cardiovascular disease. And it should be mentioned that the returns would be 1.8 times as high in the African American population according to Dehmer.[17] To simplify our QALYs/10^3-intervention calculations, we will use a larger denominator and assume interventions after 20 years old. This yields 14,300 QALYs for 5,650,000 person-years in the 100,000 cohort, and a final result of 2.5 QALYs/10^3-interventions.

Unfortunately, we did not find a manuscript with enough detail in the CPB calculation to perform the Monte Carlo simulations for these interventions.

VIII. Screening for breast, colon, and cervical cancer

Although the screening for breast, cervical, and colon cancer would not occur in the SRC, these clinics can offer the service through a partnership with their parent academic institution. For example, University of Pennsylvania's Abramson Cancer Center offers free mammography exam to patients referred by United Community Clinic. It is therefore still beneficial to calculate the impact of these referrals.

Maciosek et al. recently calculated the impact of these services.[13] They showed that 110,000 QALYs would be saved if an additional 25% of a 4,000,000 cohort are screened routinely in the 50-75 years-old range.[13] In this range there are 66,000,000 person years in the cohort,[12] and if the screening is done every 5 years, this means 13,200,000 interventions, and a total of 33 QALYs/10^3-interventions. Similar analysis of the same report yields that screening for breast cancer every two years for women in the 50-75 year range gives 17 QALYs/10^3-interventions, and 10 QALYs/10^3-interventions for the screening of cervical cancer every 3 years for women in the 20-65 age range.

References

1. Harris, R. P. *et al.* Current methods of the US Preventive Services Task Force: a review of the process. *Am. J. Prev. Med.* **20,** 21–35 (2001).
2. Taylor Jr, D. H., Hasselblad, V., Henley, S. J., Thun, M. J. & Sloan, F. A. Benefits of smoking cessation for longevity. *Am. J. Public Health* **92,** 990–996 (2002).
3. Solberg, L. I. *et al.* Tobacco use screening and counseling: technical report prepared for the National Commission on Prevention Priorities. *Minneap. MN Heal. Res. Found.* (2006).
4. Russell, M. A. H., Wilson, C., Taylor, C. & Baker, C. D. Effect of general practitioners9 advice against smoking. *Br Med J* **2,** 231–235 (1979).

5. Slama, K., Redman, S., Perkins, J., Reid, A. L. & Sanson-Fisher, R. W. The effectiveness of two smoking cessation programmes for use in general practice: a randomised clinical trial. *BMJ* **300,** 1707–1709 (1990).
6. Jayant, K., Balakrishnan, V., Sanghvi, L. D. & Jussawalla, D. J. Quantification of the role of smoking and chewing tobacco in oral, pharyngeal, and oesophageal cancers. *Br. J. Cancer* **35,** 232 (1977).
7. General, S. The health consequences of smoking—50 years of progress: a report of the surgeon general. in *US Department of Health and Human Services* (Citeseer, 2014).
8. Maciosek, M. V., Solberg, L. I., Edwards, N. M. & McGree, D. A. Alcohol Misuse Screening and Behavioral Counseling: Technical Report Prepared for the National Commission on Prevention Priorities. (2008).
9. Maciosek, M. V. *et al.* Influenza Immunization for Adults 50 Years and Older: Technical Report Prepared for the National Commission on Prevention Priorities. (2006).
10. Maciosek, M. V., Edwards, N. M., Nelson, W. W., Davis, P. M. K. & McGree, D. A. Hypertension Screening: Technical Report Prepared for the National Commission on Prevention Priorities. (2006).
11. Bedimo, A. L., Pinkerton, S. D., Cohen, D. A., Gray, B. & Farley, T. A. Condom distribution: a cost–utility analysis. *Int. J. STD AIDS* **13,** 384–392 (2002).
12. Arias, E. United States life tables, 2012. (2016).
13. Maciosek, M. V. *et al.* Updated priorities among effective clinical preventive services. *Ann. Fam. Med.* **15,** 14–22 (2017).
14. Maciosek, M. V. *et al.* Priorities among effective clinical preventive services: results of a systematic review and analysis. *Am. J. Prev. Med.* **31,** 52–61 (2006).
15. Coffield, A. B. *et al.* Priorities among recommended clinical preventive services. *Am. J. Prev. Med.* **21,** 1–9 (2001).

16. Solberg, L. I., Maciosek, M. V. & Edwards, N. M. Primary care intervention to reduce alcohol misuse: ranking its health impact and cost effectiveness. *Am. J. Prev. Med.* **34,** 143–152 (2008).

17. Dehmer, S. P., Maciosek, M. V., LaFrance, A. B. & Flottemesch, T. J. Health Benefits and Cost-Effectiveness of Asymptomatic Screening for Hypertension and High Cholesterol and Aspirin Counseling for Primary Prevention. *Ann. Fam. Med.* **15,** 23–36 (2017).

18. Oriol, N. E. *et al.* Calculating the return on investment of mobile healthcare. *BMC Med.* **7,** 27 (2009).

19. Wang, L. Y., Yang, Q., Lowry, R. & Wechsler, H. Economic Analysis of a School-Based Obesity Prevention Program. *Obesity* **11,** 1313–1324 (2003).

20. Sharma, M. School-based interventions for childhood and adolescent obesity. *Obes. Rev.* **7,** 261–269 (2006).

21. Soltani, M., Smith, S., Beck, E. & Johnson, M. Universal depression screening, diagnosis, management, and outcomes at a student-run free clinic. *Acad. Psychiatry* **39,** 259–266 (2015).

22. Chandra, A., Billioux, V. G., Copen, C. E., Balaji, A. & DiNenno, E. *HIV testing in the US household population aged 15-44: data from the National Survey of Family Growth, 2006-2010*. (US Department of Health and Human Services, Centers for Disease Control and Prevention, National Center for Health Statistics, 2012).

23. Huber, L. R. B. *et al.* Comparison of prospective and retrospective measurements of frequency of sexual intercourse. *Matern. Child Health J.* **18,** 1293–1299 (2014).

24. Molinari, N.-A. M. *et al.* The annual impact of seasonal influenza in the US: measuring disease burden and costs. *Vaccine* **25,** 5086–5096 (2007).

25. Flannery, B. & Chung, J. Influenza vaccine effectiveness, including LAIV vs IIV in children and adolescents, US Flu VE Network, 2015–16. *Advis. Comm. Immun. Pract.* **22,** (2016).

26. Fielding, J. E., Kelly, H. A., Mercer, G. N. & Glass, K. Systematic review of influenza A (H1N1) pdm09 virus shedding: duration is affected by severity, but not age. *Influenza Other Respir. Viruses* **8,** 142–150 (2014).

27. Group, C. D. C.-E. S. & others. The cost-effectiveness of screening for type 2 diabetes. *JAMA* **280,** 1757–1763 (1998).

28. Hoerger, T. J. *et al.* Screening for type 2 diabetes mellitus: a cost-effectiveness analysis. *Ann. Intern. Med.* **140,** 689–699 (2004).
