# Supplementary material for: A Monte Carlo simulation approach for estimating the health and economic impact of interventions provided at a student-run clinic
Source: PLoS One. 2017 Dec 28;12(12):e0189718. doi: 10.1371/journal.pone.0189718 (PMC5746244; doi:10.1371/journal.pone.0189718)
Supplement: S2 Appendix — (PDF) [file pone.0189718.s002.pdf]

S2: Equation flow for the clinically preventable burden (CPB) calculations for influenza vaccination of seniors

The purpose of the S2 document is to give the readers an idea of the equation flow required for CPB calculations. Going through one of these calculations also shows how the uncertainty of input parameters propagates down to the final result and why Monte Carlo simulations would be useful.

These CPB calculations were based on the preventive medicine technical report by Maciosek et al. in 2006.[1] The technical report presents detailed discussion on the input parameters (and their uncertainty) for the CPB calculations. Readers are encouraged to study this document to understand more about the choice of the CPB algorithm and the detail behind the values of the input parameters.

The python code, offered in S3 the supplementary documents of our manuscript, keeps the same notation as the ones presented here and reference [1]. The code also uses the same CPB calculation flow as reference [1] but expands by adding Monte Carlo simulations and statistical analysis from the simulations.

The S1 document also contains a discussion of the references used for all the other Monte Carlo simulations for the other interventions.

Authors who use this algorithm should also cite:

[1]: Maciosek MV, Solberg LI, Edwards NM, Khanchandani HS, Butani AL, McGree DA, et al. Influenza Immunization for Adults 50 Years and Older: Technical Report Prepared for the National Commission on Prevention Priorities. 2006.

All calculations are for a birth cohort of 4,000,000 people. Solid brackets denote the range of possible values for the input parameter due to its uncertainty. Bold script refers to calculated quantities whose uncertainties depend on the uncertainties of previous input parameters.

1) Estimate the total unadjusted influenza related deaths after age 49. “Unadjusted” refers to the fact that this is the value calculated directly from observed mortality rates.

a	Number of person-years between 50-64	a = 53,357,760
b	Number of person-years after age 64	b = 58,699,920
c	Annual influenza-related mortality rate per 100,000 (ages 50-64)	c = 12.5 [9.4 - 15.6]
d	Annual influenza-related mortality rate per	d = 132.5 [99-165]
**e**	Total unadjusted influenza related deaths after ages 49.	**e** = 84,447

$$e = \frac{(a * c + b * d)}{100{,}000}$$

*: Text was modified from reference [1] to improve clarity for broader audience.

2) Based on the rate of vaccinations, and their efficacy, calculate the theoretical mortality rates if there were no influenza vaccinations. This calculation will allow you to later estimate the QALYs gained from preventing mortalities.

f	Annual vaccination rate in ages 50-64 in 1990s*	f = 0.34 [0.26 - 0.43]
g	Annual vaccination rate in ages 65+ in 1990s*	g = 0.57 [0.43-0.72]
h	Efficacy of annual influenza vaccine in preventing influenza-related mortality	h = 0.43 [0.32-0.54]
**i**	Predicted annual influenza mortality rate per 100,000 ages 50-64 in the absence of vaccinations*	**i** = 14.6
**j**	Predicted annual influenza mortality rate per 100,000 ages 65+ in the absence of vaccinations*	**j** = 176
**k**	Predicted influenza-related mortalities after age 49 in birth cohort in the absence of vaccinations*	**k** = 111,000

$$i = \frac{c}{1 - f * h}$$

$$j = \frac{d}{1 - g * h}$$

$$k = \frac{(a * i + b * j)}{100{,}000}$$

*: Text was modified from reference [1] to improve clarity for broader audience.

3) Calculate a prediction for total incidence of influenza cases in the cohort if no individuals were routinely vaccinated. This will be useful later on when you want to calculate the QALYs gained from preventing influenza cases.

l	Annual incidence of influenza-like illness in unvaccinated individuals	l = 0.0010 [0.000 - 0.002]
**m**	Predicted number of influenza cases after age 49 if the birth cohort were unvaccinated*	**m** = 16,932,000

$$m = (a + b) * l$$

4) Similarly, calculate a prediction for the total incidence of hospitalizations caused by influenza if the cohort was not routinely vaccinated.

n	Annual hospitalization rate for pneumonia or influenza ages 50-64	n = 0.0010 [0.000-0.002]
**o**	50-64, number of hospitalizations for pneumonia in the cohort	**o** = 53,400
p	65+, Annual hospitalization rate for pneumonia in unvaccinated individuals	p = 0.009 [0.0085-0.0111]
**q**	65+, number of hospitalizations for pneumonia in the cohort	**q** = 528,000

$$\text{o} = (a * n)$$

$$\text{q} = (b * p)$$

*: Text was modified from reference [1] to improve clarity for broader audience.

5) Combine the efficacy of the vaccine and the adherence into effectiveness of the intervention.

r	Adherence with vaccine	r = 0.85 [0.75-0.95]
s	Efficacy of influenza vaccine in preventing influenza-related mortality	s = 0.43
**t**	Effectiveness of offering vaccine in preventing mortality	**t** = 0.37
u	Efficacy of vaccine in preventing influenza-like illness	u = 0.19 [0.10-0.30]
**v**	Effectiveness of offering vaccine in preventing influenza-like illness	**v** = 0.16
w	Efficacy of influenza vaccine in preventing hospitalizations	w = 0.37 [0.25-0.50]
**x**	Effectiveness of offering vaccine in preventing hospitalizations	**x** = 0.31

$$\mathrm{t} = (r * h)$$
$$\mathrm{v} = (r * u)$$
$$\mathrm{x} = (r * w)$$

*: Text was modified from reference [1] to improve clarity for broader audience.

6) Now that you have an estimate of how many people in the cohort would die of influenza if unvaccinated (*"k"*), and the effectiveness of the vaccine in preventing mortality (*"t"*), estimate the amount of mortalities prevented in the cohort if the intervention is implemented.

**y**	Predicted mortalities prevented	y = 40,477

$$y = (k * t)$$

7) Calculate the QALYs saved by preventing mortality. Use the predicted number of mortalities in each range (*a*i/100,000 and a*j/100,000*) and the average number of years lost when someone in that age group dies ("z" and "aa")

z	Average life expectancy at death ages 50-64	z = 16.4 [5.1-10.1]
aa	Average life expectancy at death ages 65+	aa = 5.6
**bb**	Years of life saved (equivalent to 1 QALY each)*	**bb** = 257,600

$$bb = t * \left[\frac{(a*i)*z}{100{,}000} + \frac{(b*j)*aa}{100{,}000}\right]$$

*: Text was modified from reference [1] to improve clarity for broader audience.

8) Similarly, calculate the QALYs gained when you prevent non-hospitalized cases of influenza. Use the predicted number of illnesses if there were no vaccinations (*"m"*) and the effectiveness of the intervention at preventing the illness ("v"). Then use the estimate that an influenza illness is a reduction of 30% in quality of life.

**cc**	Predicted non-hospitalized cases prevented	**cc** = 2,639,000
dd	Duration of illness in years*	0.0192[0.0096-0.0385]
**ee**	Year-life equivalents of illness prevented by reducing non-hospitalized cases	**ee** = 50,743
ff	QALY weight in influenza non-hospitalized cases	ff = 0.30 [0.20-0.40]
**gg**	QALYs saved due to reduced non-hospitalized cases	**gg** = 15,223

$$\text{cc} = (m * v)$$

$$\text{ee} = (cc * dd)$$

$$\text{gg} = (ee * ff)$$

*: Text was modified from reference [1] to improve clarity for broader audience.

9) Next is calculating the QALYs gained by prevented hospitalizations. Use the predicted number of hospitalizations in the absence of vaccinations (“o” and “q”) and the effectiveness of the vaccine. Also use the duration of the hospitalization and the QALY reduction.

**hh**	Predicted hospitalizations for pneumonia that would be prevented	**hh** = 180,810
ii	Duration of illness in years	ii = 0.0385[0.0189-0.058]
**jj**	Years of illness prevented by reducing hospitalizations	**jj** = 6,950
kk	QALY weight in pneumonia hospitalizations	kk = 0.30 [0.20-0.40]
**ll**	QALYs saved due to reduced hospitalizations	**ll** = 2,090

$$\text{hh} = (o + q) * ff)$$

$$\text{jj} = (hh * ii)$$

$$\text{ll} = (jj * kk)$$

10) Finally, calculate the total QALYs gained from preventing mortality, hospitalizations, and illness.

**mm**	Total QALYs saved	**mm** = 275,000

$$\text{mm} = (bb + gg + ll)$$
