# Supplementary material for: A Monte Carlo simulation approach for estimating the health and economic impact of interventions provided at a student-run clinic
Source: PLoS One. 2017 Dec 28;12(12):e0189718. doi: 10.1371/journal.pone.0189718 (PMC5746244; doi:10.1371/journal.pone.0189718)
Supplement: S3 Appendix — (ZIP) [file pone.0189718.s003.zip › S3_SecondSubmission/S3_PythonCode.pdf]

```
# -*- coding: utf-8 -*-
"""
File: Alcohol_CPB_MonteCarloSimulations.py
Final copy on Nov 19th 2017
@author: Daniel J. Arenas
Monte Carlo simulations for clinically preventable burden (QALYs gained) calculations
for alcohol misuse screening and counseling.
Although this is an open source program, researchers should cite the following papers:
Monte Carlo simulations and results:
1) "A Monte Carlo Simulation Approach for Estimating the Health and Economic Impact
of Interventions Provided at a Student-Run Clinic".
Equation flow and ranges for the input parameters are from Maciosek's publication.
Authors using this software should also cite:
2) Maciosek et al. Alcohol Misuse Screening and Be-havioral Counseling:  Technical
Report Prepared for the National Commission on Prevention Priorities. (2008).
"""
from random import *
import matplotlib.pyplot as plt
import numpy as np

La14= []
counts= 1000000                         #Number of simulations
for i in range(0,counts):
    a1 = 676632                           #a1: Alcohol-atributable life years lost to
chronic conditions
    a1 = uniform(0.80*a1, 1.20*a1)        #a1: Range: =/- 20%
    a2 = 1446836                          #a2: Alcohol-atributable life years lost to
acute conditions
    a2 = uniform(0.80*a2, 1.20*a2)        #a2: Range: =/- 20%
    a3 = 385681                           #a3: Alcohol-atributable morbidity-related
QALYs lost from chronic conditions
    a3 = uniform(0.60*a3,1.40*a3)         #a3: Range: =/- 40%
    a4 = 109120                           #a4: Alcohol-atributable morbidity-related
QALYs lost from acute conditions
    a4 = uniform(0.60*a4,1.40*a4)         #a4: Range: =/- 40%
    a5 = a1 + a2 + a3 + a4                #Total alcohol-attributable QALYs lost
    a6 = uniform(0.05,0.25)               #Delivery of screening and counseling
    a8 = uniform(0.8, 0.95)               #Adherence with screening
    a9 = uniform(0.6,0.9)                 #Average sensitivity of CAGE & AUDIT
questionnaires
    a10 = uniform(0.10,0.35)              #Effectiveness of counseling at changing
behaviour
    a11 = uniform(0.75,1)                 #Efficacy of behaviour change at reducing
chronic conditions
    a12 = uniform(0.10,0.50)              #Efficacy of behaviour change at reducing
chronic conditions
    a13 = 1/a5*(a11*(a2+a4)+a12*(a1+a3))#Weighted efficacy of behaviour change at
reducing total alcohol-attributable QALYs lost
    a7 = a5/(1-a6*a10*a13)                #Predicted alcohol-attributable QALYs lost
    a14 = a7*a8*a9*a10*a13                #QALYS gained, CPB

    #Build Lists
    La14.append(a14)


#Build Histogram
bins = np.arange(0, 1000000, 5000) # fixed bin size
plt.xlim([0, 600000])
plt.xticks(range(0,600000,100000))
plt.hist(La14, bins=bins, alpha=0.5)

#Cohort calculations Graph
plt.title('Histogram results')
plt.xlabel('Cohort: Calculated QALYs for alcohol screening and intervention')
```

```
plt.ylabel('Frequency (arb. units)')
plt.show()


#Sort and Calculate Confidence Intervals
Lsorted = sorted(La14)
lowerlimit = Lsorted[int(0.025*counts)]
upperlimit = Lsorted[int(0.975*counts)]

#Calculate Mean and Standard Deviation
print("\n\nQALYS GAINED IN COHORT")
print("Mean for QALYs gained (CPB): " + str(int(np.mean(Lsorted))) )
print("Standard deviation for QALYs gained (CPB): " + str(int(np.std(Lsorted))) )
print("Confidence interval: [" + str(int(lowerlimit)) + ", " + str(int(upperlimit)) +
"]")
print("Number of simulations: " + str(len(Lsorted)))

#Save to output file for cohort
cohortfile = open('Alcohol_Cohort_data.txt', 'w')
for item in Lsorted:
     print>>cohortfile, item
cohortfile.close()

#Read out put file from cohort
nfile = 0
with open('Alcohol_Cohort_data.txt', 'r') as inF:
    for line in inF:
       nfile = nfile + 1
cohortfile.close()
print("Number of simulation points in cohort output file: ", nfile)

#Calculate the QALYs gained by intervention
print("\n\nQALYS GAINED PER INDIVIDUAL INTERVENTION")
NI = 206000000
print("Number of interventions in the cohort: ", NI)
Lfinal = [i/NI for i in Lsorted]
lowerlimit = Lfinal[int(0.025*counts)]
upperlimit = Lfinal[int(0.975*counts)]


#Calculate Mean and Standard Deviation
print("Mean for QALYs gained (CPB): " + str((np.mean(Lfinal))) )
print("Standard deviation for QALYs gained (CPB): " + str((np.std(Lfinal))) )
print("Confidence interval: [" + str((lowerlimit)) + ", " + str((upperlimit)) + "]")
print("Number of simulations: " + str(len(Lfinal)))

#Save to output file for Intervention
indfile = open('Alcohol_Intervention_data.txt', 'w')
for item in Lfinal:
     print>>indfile, item
indfile.close()

#Read output file from individual intervention output
nfile = 0
with open('Alcohol_Intervention_data.txt', 'r') as inF:
    for line in inF:
       nfile = nfile + 1
print("Number of simulation points in intervention output file: ", nfile)


#Build Histogram and Graph
bins = np.arange(0, 0.0035, 0.000005) # fixed bin size
plt.xlim([0, 0.0035])
plt.hist(Lfinal, bins=bins, alpha=0.5)
```

```python
plt.title('Histogram results')
plt.xlabel('Calculated QALYs/intervention')
plt.ylabel('Frequency (arb. units)')
plt.show()

#==============================
#Calculate Your Clinic Impact
#==============================
intervention_mean = np.mean(Lfinal)
intervention_stdev = np.std(Lfinal)
patient_volume = 500
Fluctuation = 0.20
QALY_dollars = 132200

QALYs_year_mean = intervention_mean*patient_volume
#Var(AB)=Var(A)Var(B)+Var(A)Mean2(B)+Mean2(A)Var(B)
Term1 = (intervention_stdev*patient_volume*Fluctuation)**2
Term2 = (intervention_stdev*patient_volume)**2
Term3 = (intervention_mean*patient_volume*Fluctuation)**2

QALYs_year_stdev = (Term1 + Term2 + Term3)**0.5

print("CLINIC")
print("Alcohol Mean QALYS/year: ", QALYs_year_mean)
print("Alcohol_Stdev_QALYS/year: ", QALYs_year_stdev)
print("Monetary value (Mean): ", QALYs_year_mean*QALY_dollars)
print("Monetary value (Stdev): ", QALYs_year_stdev*QALY_dollars)
```

```
# -*- coding: utf-8 -*-
"""
File: Alcohol_CPB_MonteCarloSimulations.py
Final copy on Nov 19th 2017
@author: Daniel J. Arenas
Monte Carlo simulations for clinically preventable burden (QALYs gained) calculations
for alcohol misuse screening and counseling.
This program uses Normal distributions for the input parameters.
Although this is an open source program, researchers should cite the following papers:
Monte Carlo simulations and results:
1) "A Monte Carlo Simulation Approach for Estimating the Health and Economic Impact
of Interventions Provided at a Student-Run Clinic".
Equation flow and ranges for the input parameters are from Maciosek's publication.
Authors using this software should also cite:
2) Maciosek et al. Alcohol Misuse Screening and Be-havioral Counseling:  Technical
Report Prepared for the National Commission on Prevention Priorities. (2008).
"""
from random import *
import matplotlib.pyplot as plt
import numpy as np

La14= []
counts= 1000000                        #Number of simulations
for i in range(0,counts):
    a1 = np.random.normal(676632, 78130)                         #a1: Alcohol-
atributable life years lost to chronic conditions
    a2 = np.random.normal(1446836,167066)                        #a2: Alcohol-
atributable life years lost to acute conditions
    a3 = np.random.normal(385681,89069)                         #a3: Alcohol-
atributable morbidity-related QALYs lost from chronic conditions
    a4 = np.random.normal(109120,25200)                         #a4: Alcohol-
atributable morbidity-related QALYs lost from acute conditions
    a5 = a1 + a2 + a3 + a4             #Total alcohol-attributable QALYs lost
    a6 = np.random.normal(0.15,0.05774)            #Delivery of screening and
counseling
    a8 = np.random.normal(0.875,0.04330)            #Adherence with screening
    a9 = np.random.normal(0.75,0.0866)              #Average sensitivity of CAGE &
AUDIT questionnaires
    a10 = np.random.normal(0.225,0.07217)           #Effectiveness of counseling at
changing behaviour
    a11 = np.random.normal(0.875,0.07217)              #Efficacy of behaviour change
at reducing chronic conditions
    a12 = np.random.normal(0.3,0.11547)            #Efficacy of behaviour change at
reducing chronic conditions
    a13 = 1/a5*(a11*(a2+a4)+a12*(a1+a3))#Weighted efficacy of behaviour change at
reducing total alcohol-attributable QALYs lost
    a7 = a5/(1-a6*a10*a13)             #Predicted alcohol-attributable QALYs lost
    a14 = a7*a8*a9*a10*a13             #QALYS gained, CPB

    #Build Lists
    La14.append(a14)


#Build Histogram
bins = np.arange(0, 1000000, 5000) # fixed bin size
plt.xlim([0, 600000])
plt.xticks(range(0,600000,100000))
plt.hist(La14, bins=bins, alpha=0.5)

#Cohort calculations Graph
plt.title('Histogram results')
plt.xlabel('Cohort: Calculated QALYs for alcohol screening and intervention')
plt.ylabel('Frequency (arb. units)')
plt.show()
```

```python
#Sort and Calculate Confidence Intervals
Lsorted = sorted(La14)
lowerlimit = Lsorted[int(0.025*counts)]
upperlimit = Lsorted[int(0.975*counts)]

#Calculate Mean and Standard Deviation
print("\n\nQALYS GAINED IN COHORT")
print("Mean for QALYs gained (CPB): " + str(int(np.mean(Lsorted))) )
print("Standard deviation for QALYs gained (CPB): " + str(int(np.std(Lsorted))) )
print("Confidence interval: [" + str(int(lowerlimit)) + ", " + str(int(upperlimit)) +
"]")
print("Number of simulations: " + str(len(Lsorted)))

#Save to output file for cohort
cohortfile = open('Alcohol_Cohort_data.txt', 'w')
for item in Lsorted:
     print>>cohortfile, item
cohortfile.close()

#Read out put file from cohort
nfile = 0
with open('Alcohol_Cohort_data.txt', 'r') as inF:
    for line in inF:
       nfile = nfile + 1
cohortfile.close()
print("Number of simulation points in cohort output file: ", nfile)

#Calculate the QALYs gained by intervention
print("\n\nQALYS GAINED PER INDIVIDUAL INTERVENTION")
NI = 206000000
print("Number of interventions in the cohort: ", NI)
Lfinal = [i/NI for i in Lsorted]
lowerlimit = Lfinal[int(0.025*counts)]
upperlimit = Lfinal[int(0.975*counts)]


#Calculate Mean and Standard Deviation
print("Mean for QALYs gained (CPB): " + str((np.mean(Lfinal))) )
print("Standard deviation for QALYs gained (CPB): " + str((np.std(Lfinal))) )
print("Confidence interval: [" + str((lowerlimit)) + ", " + str((upperlimit)) + "]")
print("Number of simulations: " + str(len(Lfinal)))

#Save to output file for Intervention
indfile = open('Alcohol_Intervention_data.txt', 'w')
for item in Lfinal:
     print>>indfile, item
indfile.close()

#Read output file from individual intervention output
nfile = 0
with open('Alcohol_Intervention_data.txt', 'r') as inF:
    for line in inF:
       nfile = nfile + 1
print("Number of simulation points in intervention output file: ", nfile)


#Build Histogram and Graph
bins = np.arange(0, 0.0035, 0.000005) # fixed bin size
plt.xlim([0, 0.0035])
plt.hist(Lfinal, bins=bins, alpha=0.5)
plt.title('Histogram results')
plt.xlabel('Calculated QALYs/intervention')
```

```
plt.ylabel('Frequency (arb. units)')
plt.show()

#==============================
#Calculate Your Clinic Impact
#==============================
intervention_mean = np.mean(Lfinal)
intervention_stdev = np.std(Lfinal)
patient_volume = 500
Fluctuation = 0.20
QALY_dollars = 132200

QALYs_year_mean = intervention_mean*patient_volume
#Var(AB)=Var(A)Var(B)+Var(A)Mean2(B)+Mean2(A)Var(B)
Term1 = (intervention_stdev*patient_volume*Fluctuation)**2
Term2 = (intervention_stdev*patient_volume)**2
Term3 = (intervention_mean*patient_volume*Fluctuation)**2

QALYs_year_stdev = (Term1 + Term2 + Term3)**0.5

print("CLINIC")
print("Alcohol Mean QALYS/year: ", QALYs_year_mean)
print("Alcohol_Stdev_QALYS/year: ", QALYs_year_stdev)
print("Monetary value (Mean): ", QALYs_year_mean*QALY_dollars)
print("Monetary value (Stdev): ", QALYs_year_stdev*QALY_dollars)
```

```python
#!/usr/bin/env python2
# -*- coding: utf-8 -*-
"""
File: Clinic_Impact.py
Final copy Nov 19th 2017
@author: Daniel J. Arenas
This program takes an input file: Table_Input.data. The input file, also available in
S3, has
the QALYs/1000-intervention values and the patient volume for each intervention.
The program calculates the total QALYs gained at UCC, the mean and standard deviation,
as well as the dollars gained. Resuls are printed to screen.

The input file can easily be modified for another clinic.
"""
from random import *
import matplotlib.pyplot as plt
import numpy as np

def Variance_Multiplication(x,deltax,y,deltay):
   term1 = (deltax*deltay)**2
   term2 = (x*deltay)**2
   term3 = (deltax*y)**2
   return(term1+term2+term3)

def Impact(x,Dollars):
    q = float(x[1])
    dq = float(x[2])
    pv = float(x[3])
    dpv = float(x[4])
    qaly_mean = q*pv/1000
    qaly_var = Variance_Multiplication(q/1000,dq/1000,pv,dpv)
    dollars_mean = qaly_mean * Dollars
    dollars_var = Variance_Multiplication(qaly_mean, qaly_var**0.5, Dollars, 0)  #20%
fluctuation in dollar value of QALY
    result = [qaly_mean, qaly_var**0.5, dollars_mean/1000, (dollars_var**0.5)/1000]
#    qaly_stdev = Variance_Multiplication(intervention[1],intervention[2],intervention
[3],intervention[4])
    return(result)


#Open input file
z = []
with open('Table_Input.data', 'r') as inF:
    for line in inF:
      line = line.replace("\r", "")
      line = line.replace("\n","")
      values = line.split("\t")
      z.append(values)

#Generate Table 3: QALYs gained at UCC
total_mean = 0; total_var = 0; dollars_total = 0; dollars_total_var = 0
for i in range(0,11):
    total = Impact(z[i],132000)
    total_mean = total_mean + total[0]
    total_var = total_var + total[1]**2
    dollars_total = dollars_total + total[2]
    dollars_total_var = dollars_total_var + total[3]**2
    print(total)
print("\nCurrent interventions:")
print("QALYs(mean), (SD), Thousands of Dollars (mean), (SD))")
print([total_mean, total_var**0.5, dollars_total, dollars_total_var**0.5] )
print("\n\n")

#Generate Table 4: QALYs gained for potential interventions
```

```
total_mean = 0; total_var = 0; dollars_total = 0; dollars_total_var = 0
for i in range(11,14):
    total = Impact(z[i],132000)
    total_mean = total_mean + total[0]
    total_var = total_var + total[1]**2
    dollars_total = dollars_total + total[2]
    dollars_total_var = dollars_total_var + total[3]**2
    print(total)
print("\nPotential interventions:")
print("QALYs(mean), (SD), Thousands of Dollars (mean), (SD))")
print([total_mean, total_var**0.5, dollars_total, dollars_total_var**0.5] )
```

```python
# -*- coding: utf-8 -*-
"""
File: CondomDistribution_CPB_MonteCarlo_Simulations.py
Final copy on Nov 19th 2017
@author: Daniel J. Arenas
Monte Carlo simulations for clinically preventable burden (QALYs gained) calculations
for condom distribution.
Although this is an open source program, researchers should cite the following papers:
Monte Carlo simulations and results:
1) "A Monte Carlo Simulation Approach for Estimating the Health and Economic Impact
of Interventions Provided at a Student-Run Clinic".
Authors using this software should also cite:
2) Bedimo, Ariane Lisann et al. "Condom distribution: a cot-utility analysis."
International journal of STD & AIDS 13.6 (2002): 384-392
"""
from random import *
import matplotlib.pyplot as plt
import numpy as np
import scipy as sp
import scipy.stats as st


def P(f, alpha, alpha_prime, n, m, Pi_prime):
    """Calculate the probability that an uninfected person becomes infected
    as a result of n acts of intercourse with m partners assuming that f
    (fraction) of contacts are protected by condoms"""
    result = 1 - ( (1 - Pi_prime) + Pi_prime*(1-alpha)**(n - n*f)*(1- alpha_prime)**
(f*n))**m
    return(result)

def S(f, alpha, alpha_prime, n, m, Pi_prime):
    """Calculate the probability that an uninfected person becomes infected
    as a result of n acts of intercourse with an already infected partner"""
    result = (1-Pi_prime)*m*(1-(1-alpha)**(n - n*f)*(1-alpha_prime)**(f*n))
    return(result)

LA = []
LAQ = []
Interval = []
counts= 1000000  #Number of simulations
for i in range(0,counts):
    ############################################################################
    #WOMEN
    ############################################################################
    m = uniform(1,2)                        # Number of sex partners
    n = np.random.normal(86/m,3/m)      # Acts of intercourse per partner (3 years)
Karraker2011
    f1 = 0.28      #
    f2 = 0.36
    alpha = uniform(0.0003,0.0015)          #Probability of HIV transmission,
unprotected act
    alpha_prime = uniform(0.00005,0.0002)   #Probability of HIV transmission, condom-
protected act
    Pi = uniform(0.008, 0.032)              #Prevalence of HIV infection in study
population
    Pi_prime = uniform(0.003,0.012)         #Prevalence of HIV infection in study
partners
    N = 500                                 #Patients reached by intervention

    #Calculate the probability that an uninfected person becomes infected
    #as a result of n acts of intercourse with m partners assuming that f
    #(fracton) of contacts are protected by condoms
    P1 = P(f1, alpha, alpha_prime, n, m, Pi_prime)
    P2 = P(f2, alpha, alpha_prime, n, m, Pi_prime)
```

```
    #Calculate the probability that an uninfected person becomes infected
    #as a result of n acts of intercourse with an already infected partner"""
    S1 = S(f1, alpha, alpha_prime, n, m, Pi_prime)
    S2 = S(f2, alpha, alpha_prime, n, m, Pi_prime)
    #The total number of primary infections averted by the intervention
    Ap = (P1-P2)*(1-Pi)*N
    #The total number of secondary infections averted by the intervention
    As = (S1 - S2)*Pi*N
    #Total number of intections prevented
    AW = Ap + As
    ############################################################################
    #MEN
    ############################################################################
    m = uniform(1,2)           # Number of sex partners
    n = np.random.normal(86/m,3/m)         #Acts of intercourse per partner
    f1 = 0.40      #
    f2 = 0.52
    alpha = uniform(0.0003,0.0015)       #Probability of HIV transmission,
unprotected act
    alpha_prime = uniform(0.00005,0.0002)#Probability of HIV transmission, condom-
protected act
    Pi = uniform(0.008, 0.032)          #Prevalence of HIV infection in study
population
    Pi_prime = uniform(0.003,0.012)    #Prevalence of HIV infection in study partners
    N = 500          #Patients reached by intervention
    #Calculate the probability that an uninfected person becomes infected
    #as a result of n acts of intercourse with m partners assuming that f
    #(fracton) of contacts are protected by condoms
    P1 = P(f1, alpha, alpha_prime, n, m, Pi_prime)
    P2 = P(f2, alpha, alpha_prime, n, m, Pi_prime)
    #Calculate the probability that an uninfected person becomes infected
    #as a result of n acts of intercourse with an already infected partner"""
    S1 = S(f1, alpha, alpha_prime, n, m, Pi_prime)
    S2 = S(f2, alpha, alpha_prime, n, m, Pi_prime)
    #The total number of primary infections averted by the intervention
    Ap = (P1-P2)*(1-Pi)*N
    #The total number of secondary infections averted by the intervention
    As = (S1 - S2)*Pi*N
    #Total number of intections prevented
    AM = Ap + As
    ############################################################################
    A = AW + AM
    Q = 11.23                           #QALYs saved per prevented infection
    AQ = A*11.23                        #Total QALYs saved

    #Build Lists
    LA.append(A)                        #Q: QALYs saved per prevented infection
    LAQ.append(AQ)                      #AQ: Total QALYs saved


#Build Histogram and Graph
bins = np.arange(0, 5, 0.01) # fixed bin size
plt.xlim([0, 5])
plt.xticks(range(0,5,1))
plt.hist(LAQ, bins=bins, alpha=0.5)


#Cohort calculations Graph
plt.title('Histogram results')
plt.xlabel('Calculated QALYs for condom distribution to 1000 cohort')
plt.ylabel('Frequency (arb. units)')
plt.show()

#Sort and Calculate Confidence Intervals
```

```
Lsorted = sorted(LAQ)
lowerlimit = Lsorted[int(0.025*counts)]
upperlimit = Lsorted[int(0.975*counts)]

#Calculate Mean and Standard Deviation
print("\n\nQALYS GAINED EVERY YEAR IN COHORT")
print("Mean for QALYs gained (CPB): " + str((np.mean(Lsorted))) )
print("Standard deviation for QALYs gained (CPB): " + str((np.std(Lsorted))) )
print("Confidence interval: [" + str((lowerlimit)) + ", " + str((upperlimit)) + "]")
print("Number of simulations: " + str(len(Lsorted)))

#Save to output file for cohort
cohortfile = open('CDistribution_Cohort_data.txt', 'w')
for item in Lsorted:
     print>>cohortfile, item
cohortfile.close()

#Read out put file from cohort
nfile = 0
with open('CDistribution_Cohort_data.txt', 'r') as inF:
    for line in inF:
       nfile = nfile + 1
cohortfile.close()
print("Number of simulation points in cohort output file: ", nfile)


#Calculate the QALYs gained by intervention
print("\n\nQALYS GAINED PER INDIVIDUAL INTERVENTION")
NI = 500*12
print("Number of interventions in the cohort: ", NI)
Lfinal = [i/NI for i in Lsorted]
lowerlimit = Lfinal[int(0.025*counts)]
upperlimit = Lfinal[int(0.975*counts)]

#Calculate Mean and Standard Deviation
print("Mean for QALYs gained (CPB): " + str((np.mean(Lfinal))) )
print("Standard deviation for QALYs gained (CPB): " + str((np.std(Lfinal))) )
print("Confidence interval: [" + str((lowerlimit)) + ", " + str((upperlimit)) + "]")
print("Number of simulations: " + str(len(Lfinal)))

#Save to output file for Intervention
indfile = open('CDistribution_Intervention_data.txt', 'w')
for item in Lfinal:
     print>>indfile, item
indfile.close()

#Read output file from individual intervention output
nfile = 0
with open('CDistribution_Intervention_data.txt', 'r') as inF:
    for line in inF:
       nfile = nfile + 1
print("Number of simulation points in intervention output file: ", nfile)

#Build Histogram and Graph
bins = np.arange(0, 0.00080, 0.00001) # fixed bin size
plt.xlim([0, 0.00080])
plt.hist(Lfinal, bins=bins, alpha=0.5)
plt.title('Histogram results')
plt.xlabel('Calculated QALYs/intervention')
plt.ylabel('Frequency (arb. units)')
plt.show()

#==============================
#Calculate Your Clinic Impact
```

```python
#==============================
intervention_mean = np.mean(Lfinal)
intervention_stdev = np.std(Lfinal)
patient_volume = 500
Fluctuation = 0.20
QALY_dollars = 132200

QALYs_year_mean = intervention_mean*patient_volume
#Var(AB)=Var(A)Var(B)+Var(A)Mean2(B)+Mean2(A)Var(B)
Term1 = (intervention_stdev*patient_volume*Fluctuation)**2
Term2 = (intervention_stdev*patient_volume)**2
Term3 = (intervention_mean*patient_volume*Fluctuation)**2

QALYs_year_stdev = (Term1 + Term2 + Term3)**0.5

print("CLINIC")
print("Cdistribution Mean QALYS/year: ", QALYs_year_mean)
print("Cdistribution_Stdev_QALYS/year: ", QALYs_year_stdev)
print("Monetary value (Mean): ", QALYs_year_mean*QALY_dollars)
print("Monetary value (Stdev): ", QALYs_year_stdev*QALY_dollars)
```

```
# -*- coding: utf-8 -*-
"""
File: Hypertension_CPB_MonteCarlo_Simulations.py
Final copy on Nov 19th 2017
@author: Daniel J. Arenas
Monte Carlo simulations for clinically preventable burden (QALYs gained) calculations
for hypertension screening and treatment.
Although this is an open source program, researchers should cite the following papers:
Monte Carlo simulations and results:
1) "A Monte Carlo Simulation Approach for Estimating the Health and Economic Impact
of Interventions Provided at a Student-Run Clinic".
Equation flow and ranges for the input parameters are from Maciosek's publication.
Authors using this software should also cite:
2) Maciosek et al. Hypertension Screening:  Technical Report Prepared for the
National Commission on Prevention Priorities. (2006).
"""
from random import *
import matplotlib.pyplot as plt
import numpy as np


La63 = []
counts= 1000000     #Number of simulations
for i in range(0,counts):
    a1 = 817949                        #a1: Total CHD mortality in the birth cohort
    a1 = uniform(0.80*a1,1.20*a1)
    a2 = 96013                         #a2: Total CHF mortality in the birth cohort
    a2 = uniform(0.80*a2,1.20*a2)
    a3 = 286857                        #a3: Total stroke mortality in the birth cohort
    a3 = uniform(0.80*a3,1.20*a3)
    a4 = uniform(0.15,0.40)            #a4: % CHD mortality attributable to HTN
    a5 = uniform(0.20,0.50)            #a5: % CHF mortality attributable to HTN
    a6 = uniform(0.25,0.60)            #a6: % stroke mortality attributable to HTN
    a7 = a1*a4                         #a7: Total CHD mortality in the cohort
attributable to HTN
    a8 = a2*a5                         #a8: Total CHF mortality in the cohort
attributable to HTN
    a9 = a3*a6                         #a9: Total stroke mortality in the cohort
attributable to HTN
    a10 = uniform(0.40,0.60)           #a10: % with HTH receiving drug treatment
    a11 = uniform(0.70,0.95)           #a11: % treatment due to asymptomatic screening
    a12 = uniform(0.10,0.35)           #a12: Effectiveness of drug treatment on CHD in
clinical trials
    a13 = uniform(0.15,0.40)           #a13: Effectiveness of drug treatment on CHF in
cinical trials
    a14 = uniform(0.25,0.60)           #a14: Effectiveness of drug treatment on stroke
deaths in clinical trials
    a15 = uniform(0.7,0.9)             #a15: Adherence in clinical trials
    a16 = a7/(1-a10*a11*a12/a15)       #a16: Predicted HTN-caused CHD deaths in absence
of screening
    a17 = a8/(1-a10*a11*a13/a15)       #a17: Predicted HTN-caused CHF deaths in absence
of screening
    a18 = a9/(1-a10*a11*a14/a15)       #a18: Predicted HTN-caused stroke deaths in
absence of screening
    a19 = 3114203                      #a19: Lifetime CHD hospitalizations in cohort
    a19 = uniform(0.8*a19,1.20*a19)
    a20 = 624626                       #a20: Lifetime incidence of CHF in birth cohort
    a20 = uniform(0.8*a20,1.20*a20)
    a21 = 567842                       #a21: Lifetime incidence of strokes in birth
cohort
    a21 = uniform(0.8*a21,1.20*a21)
    a22 = a19*a4                       #a22: Lifetime incidence of HTN-caused CHD
    a23 = a20*a5                       #a23: Lifetime incidence of HTN-caused CHF
    a24 = a21*a6                       #a24: Lifetime incidence of HTN-caused strokes
```

```
    a25 = uniform(0.05,0.20)        #a25: Effectiveness of drug treatment of CHD
events in clinical trials
    a26 = uniform(0.30,0.65)        #a26: Effectiveness of drug treatment of CHF
events in clinical trials
    a27 = uniform(0.35,0.60)        #a27: Effectiveness of drug treatment of CHD
events in clinical trials
    a28 = a22/(1-a10*a11*a25/a15)   #a28: Predicted lifetime hypertension
attributable CHD hospitalizations in absence of screening
    a29 = a23/(1-a10*a11*a26/a15)   #a29: Predicted lifetime hypertension
attributable CHF hospitalizations in absence of screening
    a30 = a24/(1-a10*a11*a27/a15)   #a30: Predicted lifetime hypertension
attributable 1st strokes hospitalizations in absence of screening
    a31 = uniform(0.9,1)            #a31: %patient accepting screening
    a32 = uniform(0.7,0.95)         #a32: %patient accepting treatment
    a33 = uniform(0.3,0.5)          #a33: %patient continuing treatment
    a34 = a31*a32*a33/a15*a12       #a34: Effectiveness of screening on CHD deaths in
typical practice
    a35 = a31*a32*a33/a15*a13       #a35: Effectiveness of screening on CHF deaths in
typical practice
    a36 = a31*a32*a33/a15*a14       #a36: Effectiveness of screening on stroke deaths
in typical practice
    a37 = a31*a32*a33/a15*a25       #a37: Effectiveness of screening on CHD events in
typical practice
    a38 = a31*a32*a33/a15*a26       #a38: Effectiveness of screening on CHF events in
typical practice
    a39 = a31*a32*a33/a15*a27       #a39: Effectiveness of screening on stroke events
in typical practice
    a40 = a16*a34                   #a40: Number of CHD deaths prevented
    a41 = a17*a35                   #a41: Number of CHF deaths prevented
    a42 = a18*a36                   #a42: Number of stroke deaths prevented
    a43 = 9.1                       #a43: Average life year loss of CHD death
    a43 = uniform(0.8*a43,1.2*a43)
    a44 = 7.1
    a44 = uniform(0.8*a44,1.2*a44)  #a44: Average life year loss of CHF death
    a45 = 8.4
    a45 = uniform(0.8*a45,1.2*a45)  #a45: Average life year loss of stroke
death
    a46 = a40*a43                   #a46: Number of life years saved from CHD death
prevented
    a47 = a41*a44                   #a47: Number of life years saved from CHF death
prevented
    a48 = a42*a45                   #a48: Number of life years saved from stroke
death prevented
    a49 = a46+a47+a48               #a49: Total years of life saved
    a50 = a28*a37                   #a50: Number of NON-fatal CHD events prevented
    a51 = a29*a38                   #a51: Number of NON-fatal CHF events prevented
    a52 = a30*a39                   #a52: Number of NON-fatal stroke events prevented
    a53 = uniform(2/52,5/52)        #a53: Average duration of CHD event in years
    a54 = 2.3
    a54 = uniform(0.70*a54,1.30*a54)#a54: Average duration of CHF event in years
    a55 = 7.8
    a55 = uniform(0.70*a55,1.30*a55)#a55: Average duration of stroke event in years
    a56 = uniform(0.2,0.4)          #a56: CHD QALY reduction
    a57 = uniform(0.1,0.3)          #a57: CHF QALY reduction
    a58 = uniform(0.2,0.5)          #a58: Stroke QALY reduction
    a59 = a50*a53*a56               #a59: WALYs saved from preventing non-fatal CHD
    a60 = a51*a54*a57               #a60: QALYs saved from preventing non-fatal CHF
    a61 = a52*a55*a58               #a61: QALYs saved from preventing non-fatal stroke
    a62 = a59+a60+a61               #a62: total QALYs saved from preventing non-fatal
events
    a63 = a49+a62                   #a63: Clinically preventable burden
    La63.append(a63)
```

```
#Build Histogram and Graph
bins = np.arange(0, 1500000, 15000) # fixed bin size
plt.xlim([0, 1500000])
plt.xticks(range(0,1500000,250000))
plt.hist(La63, bins=bins, alpha=0.5)


#Cohort calculations Graph
plt.title('Histogram results')
plt.xlabel('Cohort: Calculated QALYs for Hypertension screening and intervention')
plt.ylabel('Frequency (arb. units)')
plt.show()


#Sort and Calculate Confidence Intervals
Lsorted = sorted(La63)
lowerlimit = Lsorted[int(0.025*counts)]
upperlimit = Lsorted[int(0.975*counts)]

#Calculate Mean and Standard Deviation
print("\n\nQALYS GAINED IN COHORT")
print("Mean for QALYs gained (CPB): " + str(int(np.mean(Lsorted))) )
print("Standard deviation for QALYs gained (CPB): " + str(int(np.std(Lsorted))) )
print("Confidence interval: [" + str(int(lowerlimit)) + ", " + str(int(upperlimit)) +
"]")
print("Number of simulations: " + str(len(Lsorted)))

#Save to output file for cohort
cohortfile = open('Hypertension_Cohort_data.txt', 'w')
for item in Lsorted:
     print>>cohortfile, item
cohortfile.close()

#Read out put file from cohort
nfile = 0
with open('Hypertension_Cohort_data.txt', 'r') as inF:
    for line in inF:
       nfile = nfile + 1
cohortfile.close()
print("Number of simulation points in cohort output file: ", nfile)

#Calculate the QALYs gained by intervention
print("\n\nQALYS GAINED PER INDIVIDUAL INTERVENTION")
NI = 226000000
print("Number of interventions in the cohort: ", NI)
Lfinal = [i/NI for i in Lsorted]
lowerlimit = Lfinal[int(0.025*counts)]
upperlimit = Lfinal[int(0.975*counts)]


#Calculate Mean and Standard Deviation
print("Mean for QALYs gained (CPB): " + str((np.mean(Lfinal))) )
print("Standard deviation for QALYs gained (CPB): " + str((np.std(Lfinal))) )
print("Confidence interval: [" + str((lowerlimit)) + ", " + str((upperlimit)) + "]")
print("Number of simulations: " + str(len(Lfinal)))

#Save to output file for Intervention
indfile = open('Hypertension_Intervention_data.txt', 'w')
for item in Lfinal:
     print>>indfile, item
indfile.close()

#Read output file from individual intervention output
nfile = 0
```

```
with open('Hypertension_Intervention_data.txt', 'r') as inF:
    for line in inF:
       nfile = nfile + 1
print("Number of simulation points in intervention output file: ", nfile)

#Build Histogram and Graph
bins = np.arange(0, 0.0100, 0.00001) # fixed bin size
plt.xlim([0, 0.012])
plt.hist(Lfinal, bins=bins, alpha=0.5)
plt.title('Histogram results')
plt.xlabel('Calculated QALYs/intervention')
plt.ylabel('Frequency (arb. units)')
plt.show()

#==============================
#Calculate Your Clinic Impact
#==============================
intervention_mean = np.mean(Lfinal)
intervention_stdev = np.std(Lfinal)
patient_volume = 500
Fluctuation = 0.20
QALY_dollars = 132200

QALYs_year_mean = intervention_mean*patient_volume
#Var(AB)=Var(A)Var(B)+Var(A)Mean2(B)+Mean2(A)Var(B)
Term1 = (intervention_stdev*patient_volume*Fluctuation)**2
Term2 = (intervention_stdev*patient_volume)**2
Term3 = (intervention_mean*patient_volume*Fluctuation)**2

QALYs_year_stdev = (Term1 + Term2 + Term3)**0.5

print("CLINIC")
print("HTN Mean QALYS/year: ", QALYs_year_mean)
print("HTN Stdev QALYS/year: ", QALYs_year_stdev)
print("Monetary value (Mean): ", QALYs_year_mean*QALY_dollars)
print("Monetary value (Stdev): ", QALYs_year_stdev*QALY_dollars)
```

```
 # -*- coding: utf-8 -*-
"""
File: Influenza_Adults_CPB_MonteCarlo_Simulations.py
Final Copy on Nov 19th 2017
@author: Daniel J. Arenas
Monte Carlo simulations for clinically preventable burden (QALYs gained) calculations
for influenza vaccinations in the 15-49 age range.
Although this is an open source program, researchers should cite the following papers:
Monte Carlo simulations and results:
1) "A Monte Carlo Simulation Approach for Estimating the Health and Economic Impact
of Interventions Provided at a Student-Run Clinic".
"""
from random import *
import matplotlib.pyplot as plt
import numpy as np

Lp = []
counts= 1000000
for i in range(0,counts):
    a = 1.2 * 10**8  # Number of person-years between age 18-50 for 4,000,000 cohort
    b = np.random.normal(0.066, 0.017) # Annual incidence of influenza-like illnesss
in unvaccinated individuals. Values from Molinari2007
    c = a*b                  #Total number of influenza cases
    d = 0.35           #Effectiveness of the vaccine to prevent influenza-cases
(FlanneryInfluenza2005, MaciosekTRInfluenza2006)
    e = 9 * 10**(-5)    #Probability of death after acquring influenza in the 18-49
range (Molinari2007)
    f = c*e             #Predicted influenza mortalities during 18-49 yeard old.
4,000,000 cohort.
    g = uniform(0.75,0.95)  #Adherence

    #Prevented mortality
    h = 0.35           # Effectiveness of the vaccine in preventing death
(Molinari2007)
    i = f * g * h      # Predicted-mortalities prevented
    j = uniform(25,50)             #Average life expectancy for people between 18-49
    k = j * i          #Life years = QALYs gained by preventing mortality (LY = a
full QALY)

    #Reduced hospitalized cases
    l = c * d           # Predicted number of influenza cases prevented
    m = uniform(6/365,12/365)  # Duration of hospital stay  (Fielding2014)
    n = uniform (0.20,0.40)   # QALY reduction due to influenza
    o = l * m * n             #QALYs saved by reducing hospitalizations
    p = o + k               #Total QALYs saved
    Lp.append(p)


#Build Histogram and Graph
bins = np.arange(0, 20000, 100) # fixed bin size
plt.xlim([0, 20000])
plt.hist(Lp, bins=bins, alpha=0.5)

#Cohort calculations Graph
plt.title('Histogram results')
plt.xlabel('Cohort: Calculated QALYs for Influenza vaccination (adults)')
plt.ylabel('Frequency (arb. units)')
plt.show()


#Sort and Calculate Confidence Intervals
Lsorted = sorted(Lp)
lowerlimit = Lsorted[int(0.025*counts)]
upperlimit = Lsorted[int(0.975*counts)]
```

```
#Calculate Mean and Standard Deviation
print("\n\nQALYS GAINED IN COHORT")
print("Mean for QALYs gained (CPB): " + str(int(np.mean(Lsorted))) )
print("Standard deviation for QALYs gained (CPB): " + str(int(np.std(Lsorted))) )
print("Confidence interval: [" + str(int(lowerlimit)) + ", " + str(int(upperlimit)) +
"]")
print("Number of simulations: " + str(len(Lsorted)))

#Save to output file for cohort
cohortfile = open('Influenza_Adults_Cohort_data.txt', 'w')
for item in Lsorted:
     print>>cohortfile, item
cohortfile.close()

#Read out put file from cohort
nfile = 0
with open('Influenza_Adults_Cohort_data.txt', 'r') as inF:
    for line in inF:
       nfile = nfile + 1
cohortfile.close()
print("Number of simulation points in cohort output file: ", nfile)

#Calculate the QALYs gained by intervention
print("\n\nQALYS GAINED PER INDIVIDUAL INTERVENTION")
NI = 118000000
print("Number of interventions in the cohort: ", NI)
Lfinal = [i/NI for i in Lsorted]
lowerlimit = Lfinal[int(0.025*counts)]
upperlimit = Lfinal[int(0.975*counts)]


#Calculate Mean and Standard Deviation
print("Mean for QALYs gained (CPB): " + str((np.mean(Lfinal))) )
print("Standard deviation for QALYs gained (CPB): " + str((np.std(Lfinal))) )
print("Confidence interval: [" + str((lowerlimit)) + ", " + str((upperlimit)) + "]")
print("Number of simulations: " + str(len(Lfinal)))

#Save to output file for Intervention
indfile = open('Influenza_Adults_Intervention_data.txt', 'w')
for item in Lfinal:
     print>>indfile, item
indfile.close()

#Read output file from individual intervention output
nfile = 0
with open('Influenza_Adults_Intervention_data.txt', 'r') as inF:
    for line in inF:
       nfile = nfile + 1
print("Number of simulation points in intervention output file: ", nfile)


#Build Histogram and Graph
bins = np.arange(0, 0.00020, 0.000001) # fixed bin size
plt.xlim([0, 0.00020])
plt.hist(Lfinal, bins=bins, alpha=0.5)
plt.title('Histogram results')
plt.xlabel('Calculated QALYs/intervention')
plt.ylabel('Frequency (arb. units)')
plt.show()


#==============================
#Calculate Your Clinic Impact
```

```
#==============================
intervention_mean = np.mean(Lfinal)
intervention_stdev = np.std(Lfinal)
patient_volume = 50
Fluctuation = 0.20
QALY_dollars = 132200

QALYs_year_mean = intervention_mean*patient_volume
#Var(AB)=Var(A)Var(B)+Var(A)Mean2(B)+Mean2(A)Var(B)
Term1 = (intervention_stdev*patient_volume*Fluctuation)**2
Term2 = (intervention_stdev*patient_volume)**2
Term3 = (intervention_mean*patient_volume*Fluctuation)**2

QALYs_year_stdev = (Term1 + Term2 + Term3)**0.5

print("CLINIC")
print("Flu_vaccine (adults) Mean QALYS/year: ", QALYs_year_mean)
print("Flu vaccine (adults) Stdev QALYS/year: ", QALYs_year_stdev)
print("Monetary value (Mean): ", QALYs_year_mean*QALY_dollars)
print("Monetary value (Stdev): ", QALYs_year_stdev*QALY_dollars)
```

```
# -*- coding: utf-8 -*-
"""
File: Influenza_Seniors_CPB_MonteCarlo_Simulations.py
Final copy on Nov 19th 2017
@author: Daniel J. Arenas
Monte Carlo simulations for clinically preventable burden (QALYs gained) calculations
for influenza vaccinations (seniors)
Although this is an open source program, researchers should cite the following papers:
Monte Carlo simulations and results:
1) "A Monte Carlo Simulation Approach for Estimating the Health and Economic Impact
of Interventions Provided at a Student-Run Clinic".
Authors using this software should also cite:
2) Maciosek MV et al, Influenza immunizations for adults 50 years and older:
Technical Report Prepared for the National Commission on Prevention Priorities.
(2006).
"""
from random import *
import matplotlib.pyplot as plt
import numpy as np

Ll = []
Lmm = []
counts= 1000000          #Number of simulations
for i in range(0,counts):
    a = 53357760                        #a: Number of person-years between 50-64
    b = 58699920                        #b: Number of person-years after age 64
    c = uniform(0.75*12.5,1.25*12.5)    #c: Annual influenza-related mortality rate
per 100,000 ages 50-64
    d = uniform(0.75*132.5,1.25*132.5)  #d: Annual influenza-related mortality rate
per 100,000 ages65+
    e = (a*c+b*d)/100000                #e: Total influenza related deaths after ages
49
    f = 0.342                           #f: Vaccination rate in ages 50-64 in 1990s
    f = uniform(0.75*f,1.25*f)
    g = 0.574                           #g: Vaccination rate in ages 65+ in 1990s
    g = uniform(0.75*f,1.25*f)
    h = uniform(0.35, 0.55)             #h: Efficacy of influenza vaccine in
preventing influenza-related mortality
    i = c / (1 - f*h)                   #i: Predicted annual influenza mortality rate
per 100,000 in ages 50-64 in the absence of vaccinations
    j = d / (1 - g*h)                   #j: Predicted annual influenza mortality rate
per 100,000 in ages 65+ in the absence of vaccinations
    k = 1/100000*(a*i + b*j)            #k: Predicted infuenza-related mortalities
after age 49 in birth cohort
    l = uniform(0.09,0.25)              #l: Annual incidence of influenza-like
illness in unvaccinated individuals
    m = (a+b)*l                         #m: Predicted number of influenza cases after
age 49 if the birth cohort were unvaccinated
    n = uniform(0.0001,0.0020)          #n: Annual hospitalization rate for pneumonia
or influenza ages 50-64 in unvaccinated individuals
    o = a*n                             #o: Predicted number of hospitalizations for
pneumonia or influenza ages 50-64 if the birth cohort were unvaccinated
    p = uniform(0.0085,0.0111)          #p: Annual hospitalization rate for pneumonia
or influenza ages 65+ in unvaccinated individuals
    q = b*p                             #q: Predicted number of hospitalizations for
pneumonia or influenza ages 50-64 if the birth cohort were unvaccinated
    r = uniform(0.75,0.95)              #r: Adherence with vaccine
    s = 0.429                           #s: Efficacy of vaccine in preventing
influenza-related mortality
    t = r*h                             #t: Effectiveness of OFFERING vaccine in
preventing mortality
    u = uniform(0.10,0.30)              #u: Efficacy of vaccine in preventing
influenza-like illness
    v = r*u                             #v: Effectiveness of offering vaccine in
```

```
preventing influenza-like illness
    w = uniform(0.25,0.50)              #v: Efficacy of influenza vaccine in
preveting hospitalizations for influenza and pneumonia
    x = r *w                            #x: Effectiveness of offering vaccine in
preventing hospitalizations for influenza and pneumonia
    y = k * t                           #y: Predicted mortalities prevented
    z = uniform(5.05,10.1)              #z: Average life expectancy at ages 50-64
    aa = 5.6                            #aa: Average life expectancy at 65+
    bb = t * (a*i/100000*z + b*j/100000*aa) #bb: Years of life saved
    cc  = m*v                           #cc: Predicted non-hospitalized cases
prevented
    dd = uniform(0.5/52,2/52)           #dd: Duration of illness in years
    ee = cc*dd                          #ee: Year-equivalents of illness prevented by
reducing non-hospitalized cases
    ff = uniform(0.2,0.4)               #ff: QALY reduction per year
    gg = ee*ff                          #gg: QALYs saved due to reduced non-
hospitalized cases
    hh= (o+q)*x                         #hh: Predicted hospitalizations for pneumonia
or influenza prevented
    ii = uniform(1/52,3/52)             #ii: Duration of illness (years)
    jj = hh*ii                          #jj: Year-equivalents saved by reducing
hospitalizations
    kk = uniform(0.20,0.40)             #kk: QALY reduction weight for hospitalization
    ll = jj * kk                        #ll: QALYs saved due to reduced hospitalized
cases
    mm = bb + gg + ll                   #mm: Clinically preventable burden (QALYs
saved)
    Lmm.append(mm)
    Ll.append(l)

#Build Histogram
bins = np.arange(0, 600000, 5000) # fixed bin size
plt.xlim([0, 500000])
plt.xticks(range(0,500000,100000))
plt.hist(Lmm, bins=bins, alpha=0.5)

#Cohort calculations Graph
plt.title('Histogram results')
plt.xlabel('Cohort: Calculated QALYs for influenza vaccination (seniors)')
plt.ylabel('Frequency (arb. units)')
plt.show()

#Sort and Calculate Confidence Intervals
Lsorted = sorted(Lmm)
lowerlimit = Lsorted[int(0.025*counts)]
upperlimit = Lsorted[int(0.975*counts)]

#Calculate Mean and Standard Deviation
print("\n\nQALYS GAINED IN COHORT")
print("Mean for QALYs gained (CPB): " + str(int(np.mean(Lsorted))) )
print("Standard deviation for QALYs gained (CPB): " + str(int(np.std(Lsorted))) )
print("Confidence interval: [" + str(int(lowerlimit)) + ", " + str(int(upperlimit)) +
"]")
print("Number of simulations: " + str(len(Lsorted)))

#Save to output file for cohort
cohortfile = open('Influenza50_Cohort_data.txt', 'w')
for item in Lsorted:
     print>>cohortfile, item
cohortfile.close()

#Read out put file from cohort
nfile = 0
with open('Influenza50_Cohort_data.txt', 'r') as inF:
```

```
    for line in inF:
       nfile = nfile + 1
cohortfile.close()
print("Number of simulation points in cohort output file: ", nfile)

#Calculate the QALYs gained by intervention
print("\n\nQALYS GAINED PER INDIVIDUAL INTERVENTION")
NI = 107000000
print("Number of interventions in the cohort: ", NI)
Lfinal = [i/NI for i in Lsorted]
lowerlimit = Lfinal[int(0.025*counts)]
upperlimit = Lfinal[int(0.975*counts)]


#Calculate Mean and Standard Deviation
print("Mean for QALYs gained (CPB): " + str((np.mean(Lfinal))) )
print("Standard deviation for QALYs gained (CPB): " + str((np.std(Lfinal))) )
print("Confidence interval: [" + str((lowerlimit)) + ", " + str((upperlimit)) + "]")
print("Number of simulations: " + str(len(Lfinal)))

#Save to output file for Intervention
indfile = open('Influenza50_Intervention_data.txt', 'w')
for item in Lfinal:
     print>>indfile, item
indfile.close()

#Read output file from individual intervention output
nfile = 0
with open('Influenza50_Intervention_data.txt', 'r') as inF:
    for line in inF:
       nfile = nfile + 1
print("Number of simulation points in intervention output file: ", nfile)


#Build Histogram and Graph
bins = np.arange(0, 0.0100, 0.000005) # fixed bin size
plt.xlim([0, 0.0040])
plt.hist(Lfinal, bins=bins, alpha=0.5)
plt.title('Histogram results')
plt.xlabel('Calculated QALYs/intervention')
plt.ylabel('Frequency (arb. units)')
plt.show()


#==============================
#Calculate Your Clinic Impact
#==============================
intervention_mean = np.mean(Lfinal)
intervention_stdev = np.std(Lfinal)
patient_volume = 5
Fluctuation = 0.20
QALY_dollars = 132200

QALYs_year_mean = intervention_mean*patient_volume
#Var(AB)=Var(A)Var(B)+Var(A)Mean2(B)+Mean2(A)Var(B)
Term1 = (intervention_stdev*patient_volume*Fluctuation)**2
Term2 = (intervention_stdev*patient_volume)**2
Term3 = (intervention_mean*patient_volume*Fluctuation)**2

QALYs_year_stdev = (Term1 + Term2 + Term3)**0.5

print("CLINIC")
print("Patient volume: ", patient_volume)
print("Monetary value of one QALY: ", QALY_dollars)
```

```
print("Flu_vaccine (seniors) Mean QALYS/year: ", QALYs_year_mean)
print("Flu vaccine (seniors) Stdev QALYS/year: ", QALYs_year_stdev)
print("Monetary value (Mean): ", QALYs_year_mean*QALY_dollars)
print("Monetary value (Stdev): ", QALYs_year_stdev*QALY_dollars)
```

```
# -*- coding: utf-8 -*-
"""
File: Tobacco_CPB_MonteCarlo_Simulations.py
Final copy on Nov 19th 2017
@author: Daniel J. Arenas
Monte Carlo simulations for clinically preventable burden (QALYs gained) calculations
for tobacco use screening and counseling.
Although this is an open source program, researchers should cite the following papers:
Monte Carlo simulations and results:
1) "A Monte Carlo Simulation Approach for Estimating the Health and Economic Impact
of Interventions Provided at a Student-Run Clinic".
Equation flow and ranges for the input parameters are from Solberg's publication.
Authors using this software should also cite:
2) Solberg et al, Tobacco use screening and counseling:  Technical Report Prepared
for the National Commission on Prevention Priorities. (2006).
"""
from random import *
import matplotlib.pyplot as plt
import numpy as np

Lk = []
counts= 1000000
for i in range(0,counts):
    a = uniform(1590000,2040000)        #a: Number of ever smokers in birth-cohort of
4,000,000
    b = 5.65                            #b: Average gains in life expentancy for one
quit
    b = uniform(0.75*b,1.25*b)
    c = 709063                          #c: QALYs lost to smoking atributable illness
in birth cohort
    c = uniform(0.5*c,1.5*c)
    d = c/a                             #d: QALYs lost to smoking-attributable
illnesss per smoker
    e = uniform(0.47,0.57)              #e: Portion of ever-smokers who are former
smokers
    f = uniform(0.20,0.56)              #f: Relative risk of SA disease for former
smokers compared to current ones
    g = d/(e*f +(1-e))                  #g: QALYs lost from smoking morbidity
percontinuing smoker
    h = g - g*f                         #h: QALYs saved from avoided morbidity per
smoker quit
    j = 0.030                           #j: Long-term effectiveness of repeated
counseling in inducing quits
    k = a*(b+h)*j                       #k: Clinically preventable burden (QALYs
saved)
    Lk.append(k)



#Build Histogram and Graph
bins = np.arange(0, 1000000, 2000) # fixed bin size
plt.xlim([200000, 600000 ])
plt.xticks(range(200000,600000,100000))
plt.hist(Lk, bins=bins, alpha=0.5)

#Cohort calculations Graph
plt.title('Histogram results')
plt.xlabel('Cohort: Calculated QALYs for tobacco screening and intervention')
plt.ylabel('Frequency (arb. units)')
plt.show()


#Sort and Calculate Confidence Intervals
Lsorted = sorted(Lk)
```

```
lowerlimit = Lsorted[int(0.025*counts)]
upperlimit = Lsorted[int(0.975*counts)]

#Calculate Mean and Standard Deviation
print("\n\nQALYS GAINED IN COHORT")
print("Mean for QALYs gained (CPB): " + str(int(np.mean(Lsorted))) )
print("Standard deviation for QALYs gained (CPB): " + str(int(np.std(Lsorted))) )
print("Confidence interval: [" + str(int(lowerlimit)) + ", " + str(int(upperlimit)) +
"]")
print("Number of simulations: " + str(len(Lsorted)))

#Save to output file for cohort
cohortfile = open('Tobacco_Cohort_data.txt', 'w')
for item in Lsorted:
     print>>cohortfile, item
cohortfile.close()

#Read out put file from cohort
nfile = 0
with open('Tobacco_Cohort_data.txt', 'r') as inF:
    for line in inF:
       nfile = nfile + 1
cohortfile.close()
print("Number of simulation points in cohort output file: ", nfile)

#Calculate the QALYs gained by intervention
print("\n\nQALYS GAINED PER INDIVIDUAL INTERVENTION")
NI = 226000000
print("Number of interventions in the cohort: ", NI)
Lfinal = [i/NI for i in Lsorted]
lowerlimit = Lfinal[int(0.025*counts)]
upperlimit = Lfinal[int(0.975*counts)]


#Calculate Mean and Standard Deviation
print("Mean for QALYs gained (CPB): " + str((np.mean(Lfinal))) )
print("Standard deviation for QALYs gained (CPB): " + str((np.std(Lfinal))) )
print("Confidence interval: [" + str((lowerlimit)) + ", " + str((upperlimit)) + "]")
print("Number of simulations: " + str(len(Lfinal)))

#Save to output file for Intervention
indfile = open('Tobacco_Intervention_data.txt', 'w')
for item in Lfinal:
     print>>indfile, item
indfile.close()

#Read output file from individual intervention output
nfile = 0
with open('Tobacco_Intervention_data.txt', 'r') as inF:
    for line in inF:
       nfile = nfile + 1
print("Number of simulation points in intervention output file: ", nfile)


#Build Histogram and Graph
bins = np.arange(0, 0.0100, 0.000005) # fixed bin size
plt.xlim([0, 0.0025])
plt.hist(Lfinal, bins=bins, alpha=0.5)
plt.title('Histogram results')
plt.xlabel('Calculated QALYs/intervention')
plt.ylabel('Frequency (arb. units)')
plt.show()

#==============================
```

```python
#Calculate Your Clinic Impact
#=============================
intervention_mean = np.mean(Lfinal)
intervention_stdev = np.std(Lfinal)
patient_volume = 500
Fluctuation = 0.20
QALY_dollars = 132200

QALYs_year_mean = intervention_mean*patient_volume
#Var(AB)=Var(A)Var(B)+Var(A)Mean2(B)+Mean2(A)Var(B)
Term1 = (intervention_stdev*patient_volume*Fluctuation)**2
Term2 = (intervention_stdev*patient_volume)**2
Term3 = (intervention_mean*patient_volume*Fluctuation)**2

QALYs_year_stdev = (Term1 + Term2 + Term3)**0.5

print("CLINIC")
print("Patient volume: ", patient_volume)
print("Monetary value of one QALY: ", QALY_dollars)
print("Tobacco Mean QALYS/year: ", QALYs_year_mean)
print("Tobacco Stdev QALYS/year: ", QALYs_year_stdev)
print("Monetary value (Mean): ", QALYs_year_mean*QALY_dollars)
print("Monetary value (Stdev): ", QALYs_year_stdev*QALY_dollars)
```

```
# -*- coding: utf-8 -*-
"""
File: Tobacco_CPB_Normal_Input.py
Final Copy on Nov 19th of 2017
@author: Daniel J. Arenas
Monte Carlo simulations for clinically preventable burden (QALYs gained) calculations
for tobacco use screening and counseling.
This simulation uses normal distributions for the input parameters.
Although this is an open source program, researchers should cite the following papers:
Monte Carlo simulations and results:
1) "A Monte Carlo Simulation Approach for Estimating the Health and Economic Impact
of Interventions Provided at a Student-Run Clinic".
Equation flow and ranges for the input parameters are from Solberg's publication.
Authors using this software should also cite:
2) Solberg et al, Tobacco use screening and counseling:  Technical Report Prepared
for the National Commission on Prevention Priorities. (2006).
"""
from random import *
import matplotlib.pyplot as plt
import numpy as np

Lk = []
counts= 1000000
for i in range(0,counts):
    a = np.random.normal(1815000,129904)        #a: Number of ever smokers in birth-
cohort of 4,000,000
    b = np.random.normal(5.65,0.815)            #b: Average gains in life expentancy
for one quit
    c = np.random.normal(709063, 204688)                         #c: QALYs lost to
smoking atributable illness in birth cohort
    d = c/a                             #d: QALYs lost to smoking-attributable
illnesss per smoker
    e = np.random.normal(0.52,0.0289)               #e: Portion of ever-smokers who
are former smokers
    f = np.random.normal(0.38,0.104)               #f: Relative risk of SA disease for
former smokers compared to current ones
    g = d/(e*f +(1-e))                  #g: QALYs lost from smoking morbidity
percontinuing smoker
    h = g - g*f                         #h: QALYs saved from avoided morbidity per
smoker quit
    j = 0.030                           #j: Long-term effectiveness of repeated
counseling in inducing quits
    k = a*(b+h)*j                       #k: Clinically preventable burden (QALYs
saved)
    Lk.append(k)



#Build Histogram and Graph
bins = np.arange(0, 1000000, 2000) # fixed bin size
plt.xlim([200000, 600000 ])
plt.xticks(range(200000,600000,100000))
plt.hist(Lk, bins=bins, alpha=0.5)

#Cohort calculations Graph
plt.title('Histogram results')
plt.xlabel('Cohort: Calculated QALYs for tobacco screening and intervention')
plt.ylabel('Frequency (arb. units)')
plt.show()


#Sort and Calculate Confidence Intervals
Lsorted = sorted(Lk)
lowerlimit = Lsorted[int(0.025*counts)]
```

```
upperlimit = Lsorted[int(0.975*counts)]

#Calculate Mean and Standard Deviation
print("\n\nQALYS GAINED IN COHORT")
print("Mean for QALYs gained (CPB): " + str(int(np.mean(Lsorted))) )
print("Standard deviation for QALYs gained (CPB): " + str(int(np.std(Lsorted))) )
print("Confidence interval: [" + str(int(lowerlimit)) + ", " + str(int(upperlimit)) +
"]")
print("Number of simulations: " + str(len(Lsorted)))

#Save to output file for cohort
cohortfile = open('Tobacco_Cohort_data.txt', 'w')
for item in Lsorted:
     print>>cohortfile, item
cohortfile.close()

#Read out put file from cohort
nfile = 0
with open('Tobacco_Cohort_data.txt', 'r') as inF:
    for line in inF:
       nfile = nfile + 1
cohortfile.close()
print("Number of simulation points in cohort output file: ", nfile)

#Calculate the QALYs gained by intervention
print("\n\nQALYS GAINED PER INDIVIDUAL INTERVENTION")
NI = 226000000
print("Number of interventions in the cohort: ", NI)
Lfinal = [i/NI for i in Lsorted]
lowerlimit = Lfinal[int(0.025*counts)]
upperlimit = Lfinal[int(0.975*counts)]


#Calculate Mean and Standard Deviation
print("Mean for QALYs gained (CPB): " + str((np.mean(Lfinal))) )
print("Standard deviation for QALYs gained (CPB): " + str((np.std(Lfinal))) )
print("Confidence interval: [" + str((lowerlimit)) + ", " + str((upperlimit)) + "]")
print("Number of simulations: " + str(len(Lfinal)))

#Save to output file for Intervention
indfile = open('Tobacco_Intervention_data.txt', 'w')
for item in Lfinal:
     print>>indfile, item
indfile.close()

#Read output file from individual intervention output
nfile = 0
with open('Tobacco_Intervention_data.txt', 'r') as inF:
    for line in inF:
       nfile = nfile + 1
print("Number of simulation points in intervention output file: ", nfile)


#Build Histogram and Graph
bins = np.arange(0, 0.0100, 0.000005) # fixed bin size
plt.xlim([0, 0.0025])
plt.hist(Lfinal, bins=bins, alpha=0.5)
plt.title('Histogram results')
plt.xlabel('Calculated QALYs/intervention')
plt.ylabel('Frequency (arb. units)')
plt.show()

#=============================
#Calculate Your Clinic Impact
```

```
#==============================
intervention_mean = np.mean(Lfinal)
intervention_stdev = np.std(Lfinal)
patient_volume = 500
Fluctuation = 0.20
QALY_dollars = 132200

QALYs_year_mean = intervention_mean*patient_volume
#Var(AB)=Var(A)Var(B)+Var(A)Mean2(B)+Mean2(A)Var(B)
Term1 = (intervention_stdev*patient_volume*Fluctuation)**2
Term2 = (intervention_stdev*patient_volume)**2
Term3 = (intervention_mean*patient_volume*Fluctuation)**2

QALYs_year_stdev = (Term1 + Term2 + Term3)**0.5

print("CLINIC")
print("Patient volume: ", patient_volume)
print("Monetary value of one QALY: ", QALY_dollars)
print("Tobacco Mean QALYS/year: ", QALYs_year_mean)
print("Tobacco Stdev QALYS/year: ", QALYs_year_stdev)
print("Monetary value (Mean): ", QALYs_year_mean*QALY_dollars)
print("Monetary value (Stdev): ", QALYs_year_stdev*QALY_dollars)
```
