# Supplementary material for: A Monte Carlo simulation approach for estimating the health and economic impact of interventions provided at a student-run clinic
Source: PLoS One. 2017 Dec 28;12(12):e0189718. doi: 10.1371/journal.pone.0189718 (PMC5746244; doi:10.1371/journal.pone.0189718)
Supplement: S4 Appendix — (ZIP) [file pone.0189718.s004.zip › S4_Convergence/S4_ConvergenceStudy.pdf]

S4: Convergence of CPB calculations versus the number of Monte Carlo simulations.

In our Monte Carlo approach, we used a total of 10^6 simulations of clinically preventable burden calculations to calculate a distribution of final values. From this distribution of 10^6 final values, we were able to obtain a mean, a standard deviation, and quantile intervals of the final result: QALYs/10^3-interventions.

The choice for the number of simulations was based on convergence considerations. To test the convergence, we calculated the mean and standard deviation of the kernel densities for a broad array of simulation numbers. Figure S4.1 shows the results for the hypertension CPB calculation. Both the average and the standard deviation of the distribution of QALYs/10^3-interventions values were investigated. It is clear that by 10^4 simulations the calculated results have converged well within 1%, and that 10^6 is a sufficient number of simulations. We show the results for hypertension, since these calculations were the most complex in our work (See S1 and S3 for more information).

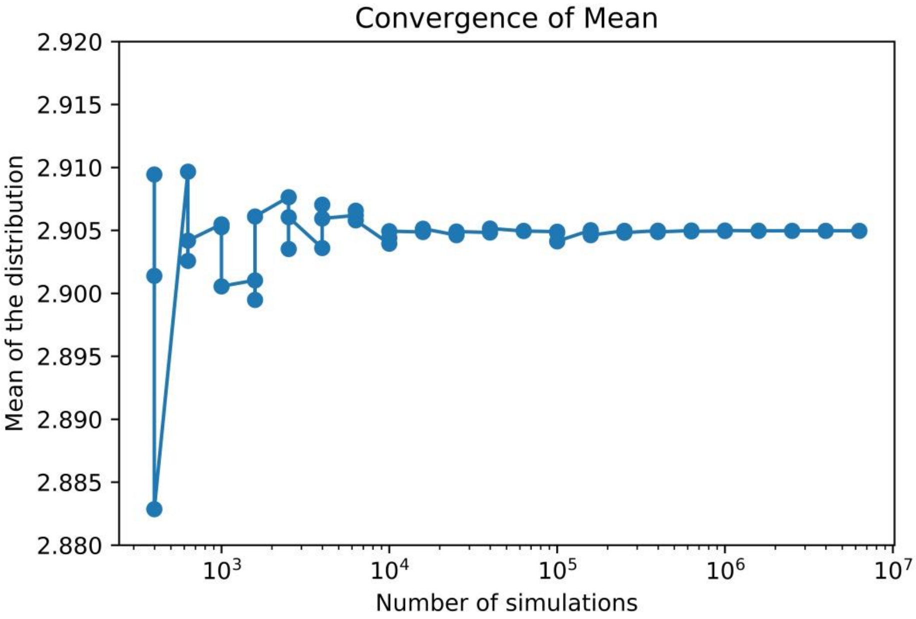

Figure S4.1 Measure of the convergence for Monte Carlo simulations of the hypertension intervention. The *x*-axis denote the number of ran simulations, and the *y*-axis denotes the mean of the distribution of QALYs/10^3-interventions values.

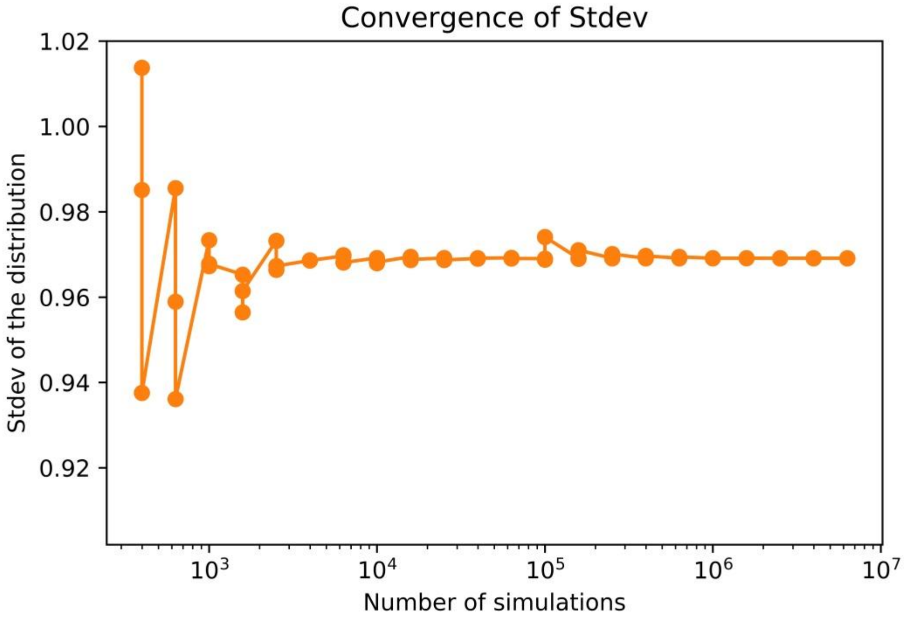

Figure S4.2 This figures shows the convergence of the standard deviation of the distribution of values of QALYs/10^3-interventions obtained from the simulations.
